# Reference genome of wild goat (*capra aegagrus*) and sequencing of goat breeds provide insight into genic basis of goat domestication

**DOI:** 10.1186/s12864-015-1606-1

**Published:** 2015-06-05

**Authors:** Yang Dong, Xiaolei Zhang, Min Xie, Babak Arefnezhad, Zongji Wang, Wenliang Wang, Shaohong Feng, Guodong Huang, Rui Guan, Wenjing Shen, Rowan Bunch, Russell McCulloch, Qiye Li, Bo Li, Guojie Zhang, Xun Xu, James W. Kijas, Ghasem Hosseini Salekdeh, Wen Wang, Yu Jiang

**Affiliations:** Kunming University of Science and Technology, Kunming, 650093 China; CAS-Max Planck Junior Research Group, State Key Laboratory of Genetic Resources and Evolution, Kunming Institute of Zoology, Chinese Academy of Sciences (CAS), Kunming, Yunnan 650223 China; BGI-Shenzhen, Shenzhen, 518083 China; Agricultural Biotechnology Research Institute of Iran, Karaj, Iran; School of Bioscience and Bioengineering, South China University of Technology, Guangzhou, 510006 China; CSIRO, Agriculture Flagship, Brisbane, 4065 QLD Australia; Centre for GeoGenetics, Natural History Museum of Denmark, University of Copenhagen, Copenhagen, Denmark; Centre for Social Evolution, Department of Biology, University of Copenhagen, Copenhagen, Denmark; Department of Molecular Systems Biology at Cell Science Research Center, Royan Institute for Stem Cell Biology and Technology, ACECR, Tehran, Iran; College of Animal Science and Technology, Northwest A&F University, Yangling, 712100 China

**Keywords:** Wild goat genome, Animal domestication, Artificial selection, Coat color evolution, Behavioral evolution

## Abstract

**Background:**

Domestic goats (*Capra hircus*) have been selected to play an essential role in agricultural production systems, since being domesticated from their wild progenitor, bezoar (*Capra aegagrus*). A detailed understanding of the genetic consequences imparted by the domestication process remains a key goal of evolutionary genomics.

**Results:**

We constructed the reference genome of bezoar and sequenced representative breeds of domestic goats to search for genomic changes that likely have accompanied goat domestication and breed formation. Thirteen copy number variation genes associated with coat color were identified in domestic goats, among which *ASIP* gene duplication contributes to the generation of light coat-color phenotype in domestic goats. Analysis of rapidly evolving genes identified genic changes underlying behavior-related traits, immune response and production-related traits.

**Conclusion:**

Based on the comparison studies of copy number variation genes and rapidly evolving genes between wild and domestic goat, our findings and methodology shed light on the genetic mechanism of animal domestication and will facilitate future goat breeding.

**Electronic supplementary material:**

The online version of this article (doi:10.1186/s12864-015-1606-1) contains supplementary material, which is available to authorized users.

## Background

The domestication of crops and livestock played a key role in the development of human society. It provides most of our food today and is even considered as an integral part of the rise of agriculture and human civilization. Understanding the domestication process has been intriguing to biologists at least since Darwin, who emphasized the wide-ranging phenotypic variations arising from domestication in his *On the Origin of Species* [1] and later sought to explain the possible causes in *The Variation of Animals and Plants Under Domestication* [2]. As for contemporary biological research, researchers have endeavored to identify genes underlying phenotypic differences associated with domestication via comparison of domesticated species and their wild ancestors [3, 4].

As one of the oldest domesticated livestock, domestic goats (*C. hircus*) are widely reared throughout the world due to their important role in agriculture, economy and culture since the Neolithic agricultural revolution [5]. The modern domestic goat was initially domesticated from the bezoar (*C. aegagrus*), in the Fertile Crescent [5]. Over the 10,000 years of domestication history, domestic goat breeds have evolved to display radically different phenotypic characteristics when compared to wild goats, ranging from physical appearances to behaviors [6]. Domestic goats are distinguished from the bezoar in a number of ways that include (1) a reduction in body size that includes smaller horns and a number of polled breeds (that lack horns); (2) increased docility; (3) a spectrum of coat color variants between breeds; (4) adaptivity with a geographically widespread distribution resulting from human migration and colonization [6–8]. The genetic bases for these acquired characteristics remain largely unknown. High quality domestic and wild goat reference genomes together with population genomic data will facilitate understanding the genetic and genic mechanisms of goat domestication, especially for selected regions with segmental duplications/deletions and rapidly evolving sequences.

## Materials and methods

### Genome sequencing and assembly

Genomic DNA from a male bezoar (*Capra aegagrus*, BioSample number is SAMN03282421) was used to construct short-insert paired-end sequencing libraries and mate pair libraries (insert sizes ranged between 250 bp to 5 kb). Due to the increased DNA requirements of longer-insert libraries, we used DNA from three additional wild goats to construct mate pair sequencing libraries (10 kb and 20 kb). Each library was sequenced with Illumina Hiseq 2000 instruments at BGI-Shenzhen. The assembly was performed using SOAPdenovo [9]. Short-insert paired-end reads were first used to construct contigs. All usable reads (from short-insert and mate-pair libraries) were then aligned to the contigs to construct scaffolds and close gaps (see the Additional file 1: Supplementary Methods 3.1 for further details).

### Anchoring scaffolds to chromosomes

To anchor wild goat scaffolds onto chromosomes, we exploited their synteny relationship with domestic goat chromosomes (CHIR_1.0). LASTZ [10] was used to align repeat-soft-masked scaffolds (longer than 2 kb) of wild goat to repeat-soft-masked chromosomes of domestic goat. We clustered the LASTZ hits within windows of 100 Kb and filtered orphan hit in each window. A wild goat scaffold could be aligned to multiple domestic goat loci or chromosomes. We sorted the alignments by length. When the longest alignment is more than twice of the second one in length, we consider it as the best alignment of this scaffold and link the wild goat’s scaffold with the aligned loci in the domestic goat’s chromosome. At last, we ordered and oriented all the linked wild goat scaffolds and constructed pseudo-chromosomes.

The wild goat Y chromosome was constructed using the following pipeline. First, BLAT [11] was used to align the bovine proteins in bovine Y chromosome (NC_016145.1 in Btau_4.6.1) to unanchored wild goat scaffolds and all the wild goat scaffolds, respectively. We filtered the low coverage (<60 % coverage of the protein length) hits. Then, we mapped contigs of bovine Y chromosome to unanchored wild goat scaffolds and all the wild goat scaffolds respectively with LASTZ. LASTZ hits were clustered based on the following standards: the distance between two hits is less than 1 Kb; or the distance is more than 1 Kb and less than 5 Kb with the unmapped region having more than 50 % repeat sequences. Combined the result of BLAT and LASTZ, we sorted the alignments by length, and picked the longest alignment of each wild scaffold and linked them to bovine chromosome Y. Using the alignment order of bovine Y chromosome, we constructed the draft assembly of wild goat chromosome Y (see the Additional file 1: Supplementary Methods 3.1 for further details).

### Annotation

Repeats in the wild goat genome assembly were first identified using RepeatMasker (http://repeatmasker.org). We then used ab initio prediction, homologous proteins alignment, RNA-seq data and EST data of domestic goat to annotate protein-coding genes in wild goat genome, building a consensus gene set by merging all the evidence mentioned above. Firstly, we used the ab initio gene prediction methods AUGUSTUS [12], GENSCAN [13] and GlimmerHMM [14, 15] to predict protein-coding genes. For homology-based protein alignment, we aligned protein sequences of six related species to the repeat-masked wild goat genome using BLAT [11] for fast alignment and then Genewise [16] for accurate gene structure. The final gene set was generated by merging all the resources with GLEAN [17]. Gene functions were assigned according to the best hit of the alignment using Blastp [18] to the SwissProt and TrEMBL databases [19]. The motifs and domains of wild goat genes were determined by InterProScan [20] against publicly available protein databases, including ProDom, PRINTS, Pfam, SMART, PANTHER and PROSITE. Gene Ontology (GO) [21] IDs for each gene were obtained from the corresponding relationship between InterPro entries and GO entries. All of the wild goat genes were aligned against KEGG [22] proteins using Blastp to predict the pathway in which the genes might be involved.

### Gene family analysis

The protein-coding genes from 7 mammalian species (*C. hircus*, *O. aries*, *B. taurus*, *S. scrofa*, *E. caballus*, *C. familiaris* and *H.sapiens*) in addition to wild goat genes were used to define gene families that descended from a single gene in the last common ancestor by Treefam [23]. Only the longest isoform for each gene was kept, and only proteins longer than 30 amino acids were retained. Then single-copy genes obtained from this analysis were used to reconstruct phylogenies by MrBayes [24] and PhyML [25] and estimate the times since divergence by PAML [26]. To identify gene families that had undergone expansion or contraction, we applied the Computational Analysis of gene Family Evolution (CAFÉ) program [24] to infer the rate and direction of change in gene family size over a given phylogeny.

### Heterozygosity analysis

We used the BWA [27] program to map the ~10 X filtered reads from the wild goat (the wild Bamu goat) and the reference domestic goat (the Yunnan black goat, BioSample number is SAMN02953816) onto their respective reference genome with default parameters. Sorting and de-duplication of the mapping results were then performed by SAMtools pipeline [28]. SOAPsnp was used to call SNP using parameters “-q –Q –L 150”. A total of 4,192,942 heterozygous SNPs were called in wild goat sequencing reads and the heterozygosity of wild goat was calculated as 0.160 %. And, a total of 4,004,154 heterozygous SNPs were called in the reference domestic goat genome and the heterozygosity of domestic goat was calculated as 0.167 %.

### CNV analysis and validation

Copy number variants were identified using wild goat genome as reference. Paired-end reads were aligned onto unmasked scaffolds of wild goat by BWA [27] with default parameters. We used the CNV calling pipeline based on the previous study [29] with a small change. We counted the aligned read numbers for every chromosome in 200 bp sliding windows with 100 bp slide steps by self-made perl script. If five out of seven or more sequential 200 bp overlapping windows in a region had values of read depth that were significantly different from the mean depth in that chromosomes (more than mean + 2 standard deviations), the region was defined as CNV gain region. CNV loss windows were initially defined as 200 bp windows with very low read depth (<0.1 average whole-genome depth), within which at least one other individual showed normal read depth (>0.5 average whole-genome depth). Five or more such 200 bp overlapping windows out of seven sequential 200 bp windows are difined as CNV loss regions. We then investigate candidate genes with significant copy number changes (genes with single copy in one species but with multi-copies in the other species) between wild goat and domestic goat. Candidate deleted genes in domestic goat was identified based on two standards: (1) if a gene mapping to a CNV region with CNV ratio (Read depth (RD)/one average fold depth in reference, e.g. if CNV ratios fluctuating around two in a region means the region has two copies in the individual compared with reference.) < 0.2 in all domestic samples but CNV ratio > =0.5 in the two wild goat samples; (2) if no significant blastp hit (Evalue < 10^−5^ and identity > =95 %) was found in domestic goat genome. Likewise, we also identified candidate duplicated genes in domestic goat.

Quantitative real-time PCR was performed for CNV validation using the QuantStudio™ 12 K Flex Real-Time PCR System. Two to Three primer pairs were respectively designed for five genic CNVs. Finally, the validated primers (Additional file 1: Table S22) by standard curve analysis using a serial dilution of genomic DNA were used for further validation experiments. An averageΔCt for each DNA sample was calculated after normalizing to *C7orf28b*, a single copy gene in mammals.

### Rapidly evolving gene analysis

We used PAML package [26] to identify lineage-specific rapidly evolving genes in wild goat and domestic goat by estimating the omega ratio (ω) of non-synonymous substitutions to synonymous substitutions to examine the selective constraints on candidate genes. Using a simplified Treefam pipeline [23], 11,847 1:1:1:1 orthologous genes in wild goat, domestic goat, cattle and human were identified, whose coding sequence was then aligned using PRANK [30] with default parameters. We estimated the ω ratio for the 11,847 orthologous genes in four species by specifying either wild goat or domestic goat as foreground branch. Considering that not all sites in nucleotide sequence have identical selection pressure, we also used branch-site model of PAML to detect positive selected sites in genes. Genes having both elevated ω and positive selected sites were then defined as positive selected candidate genes (PSGs). To further filter out the false positive results, we then check non-synomous mutations in the PRANK alignment result to exclude false positive calling induced by gaps and misalignment.

### Data access

This wild goat genome assembly has been deposited on GenBank, CapAeg_1.0 (GCA_000978405.1). Raw sequence data is available under the accession No. SRA184825. The five domestic goat Genome data were submitted to the EBI ENA under accession number ERA242189. All of the wild assembly data and its coordinate gene annotation data were also deposited in our goat genome database, http://caprinae.kiz.ac.cn.

## Results and discussion

### *De novo* assembly bezoar genome and data generation

The reference genome of the wild goat (*C. aegagrus*) was constructed using second-generation sequencing Illumina platform. Short-insert paired-end libraries and mate pair libraries with insert size from 250 bp to 5 kbp were constructed from DNA of a male wild goat (collected from Bamu of Iran) and sequenced to generate approximately 108.5-fold sequence coverage of data. Due to the increased DNA requirements of longer-insert libraries, three additional male wild goats were used to generate 27.7-fold sequence coverage of data from long-insert libraries (10 – 20 kbp) required for the scaffolding process (Additional file 1: Table S1 and Figure S1). In total, 381.50 Gb of raw sequencing data with 136.26-fold sequence coverage were generated. *De novo* assembly of the short reads using SOAPdenovo software [9] generated a draft assembly, CapAeg_1.0, with contig N50 size of 18,965 bp and scaffold N50 size of 2,057,686 bp. 23,217 genes were annotated in the wild goat genome (Additional file 1: Table S2-S10 and Figure S2-S10). Assembly data QC and read trimming can be found in Additional file 1: Supplementary Method.

We anchored ~ 90.7 % total length of scaffolds into pseudo-chromosomes based on domestic goat’s autosomes, X chromosome [31] and bovine Y chromosome (GenBank accession NO.CM001061) (Additional file 1: Table S15). The assembled wild goat Y chromosome represents the first goat Y chromosome assembly and is approximately 17.3 Mb in length with 79 anchored scaffolds. We annotated 57 genes on the Y scaffolds, of which 11 are known male specific region (MSY) genes (Additional file 1: Table S16).

**Fig. 1:**
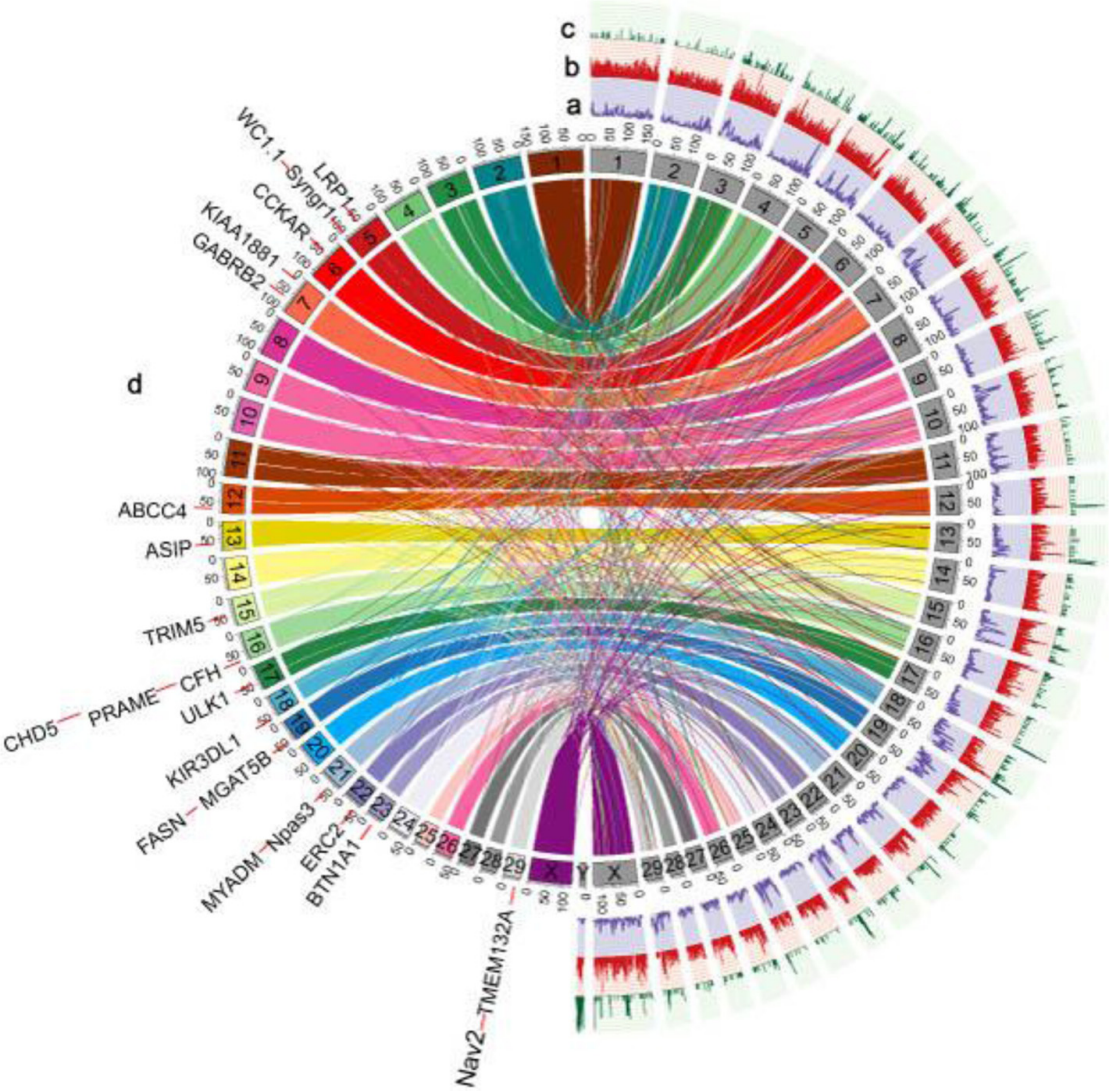
Genomic synteny of assembled chromosomes between wild goat (right half) and domestic goat (left half). In the right half, (a) distribution of gene counts in 1-Mb non-overlapping windows; (b) Distribution of CNV gain region (>800 bp in domestic goats using wild goat as reference) counts in 1-Mb non-overlapping windows; (c) Distribution of CNV loss region counts in 1-Mb non-overlapping windows. (d) In the left half, candidate domestication loci in domestic goat genome

Comparison of the wild goat assembly against the reference genome of domestic goat [31] revealed that the wild goat has higher sequence coverage, and slightly superior assembly statistics (Fig. 1) (Additional file 1: Table S4). We identified 18 positively selected candidate genes (PSGs, passing both branch model test and branch-site model test with *P* < 0.05) in the wild goat and 52 in the domestic goat by using human and cattle as out-groups (Additional file 1: Table S12 and S13; Additional file 2). The proportions of heterozygous sites within the reference genome of the wild goat (0.160 %) and domestic goat genome (0.167 %) were not significantly different. This likely suggests that the population bottleneck associated with the domestication process was not as severe as for other domesticated species [32, 33].

**Fig. 2:**
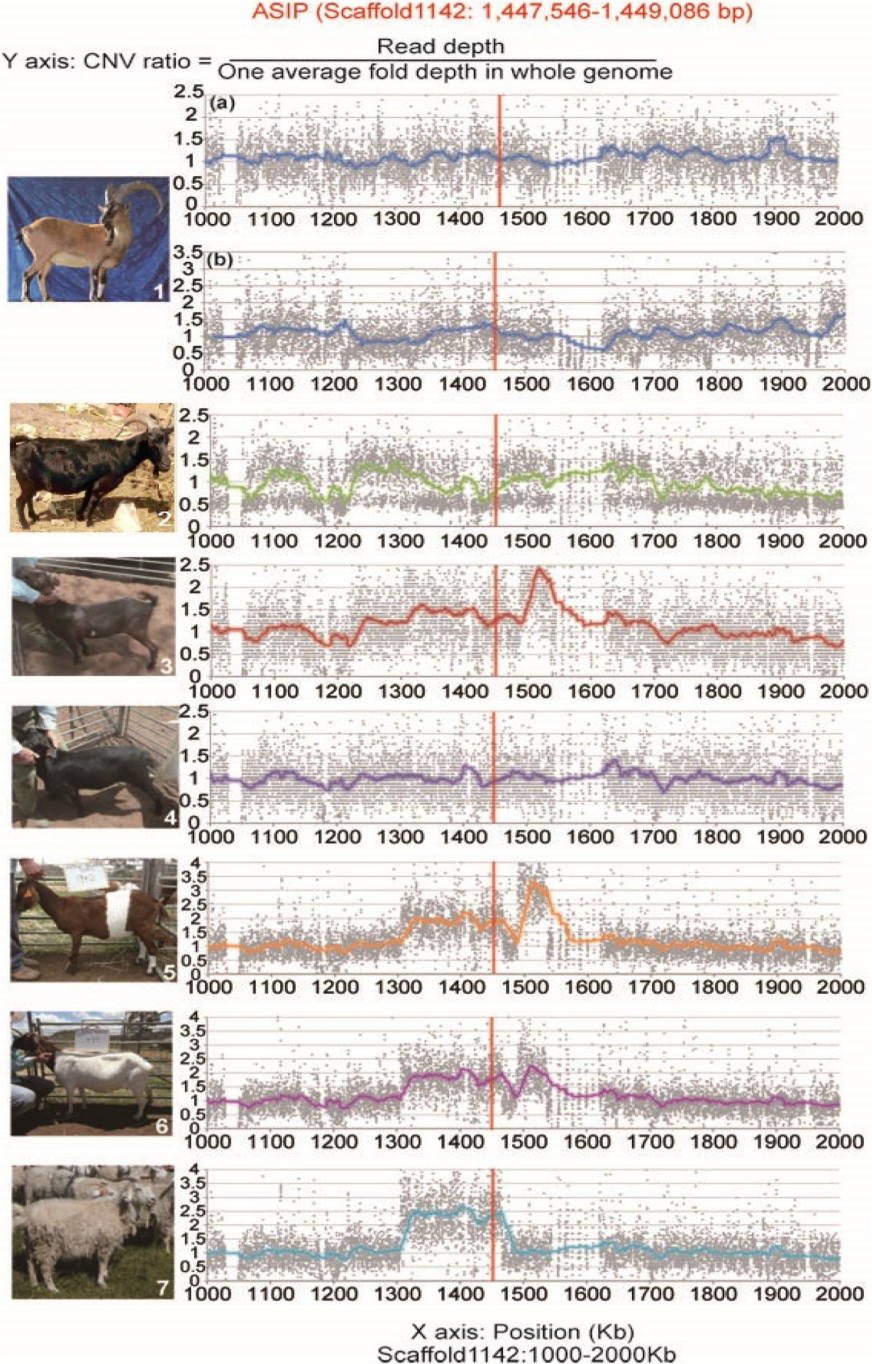
Photos of wild goat and re-sequenced domestic goat breeds with CNV ratio curve around the *ASIP* gene region. The CNV ratios (CNV ratio = Read Depth/One average fold depth in whole genome, e.g. CNV ratios fluctuating around two means the read depth of the region is two-fold of the mean in whole genome) calculated within 200-bp sliding windows with 100-bp slide steps were shown as scatter plot and fitted with moving average trend line. Around *ASIP* (wild goat scaffold1142:1,447,546-1,449,086 bp) region, Bamu wild goat, Khonj wild goat, Yunnan black goat and two Rangeland goats were detected as having one copy while two Boer goats and a Cashmere goat were detected as having at least two copies. Panel 1: wild goat (tan) (Photo is provided by Ghasem Hosseini Salekdeh.); (**a**) Bamu wild goat; (**b**) Khonj wild goat. Panel 2: Yunnan Black goat (black); Panel 3–4: Rangeland goats (dark brown and black); Panel 5–6: Boer goats (banded and Schwartzal); Panel 7: Cashmere goat (white) (Photos of Panel 3–7 are provided by James Kijas)

Gene copy number variation (CNV) is a well-established cause of gene family member differentiation and a common mechanism underpinning evolutionary change. We evaluated its contribution to the evolution of domestic goat by comparison of the wild goat assembly with re-sequenced genomes from a selection of representative domestic goat breeds. Sequencing data from the Bamu wild goat and one of the three additional male wild goats – Khonj wild goat for which we collected more sequencing data were extracted and used as wild goat individuals’ sequencing data in the CNV analysis. Six fold to eight fold whole genome resequencing was performed for two Australian feral Rangeland goats, two Boer goats and one Australian Cashmere goat (pictures of each breed shown in Fig. 2, detailed sample information and sequencing statistics shown in Additional file 1: Table S17). Combining the previously published Yunnan Black goat genome data [31] and two wild goat individuals sequencing data extracted from our *de novo* sequencing libraries, we used six domestic goats and two wild goats’ sequencing data to identify high-confidence CNVs. A total of 13,347 CNVs were called containing 1,584 genes including 9,650 CNV gain regions (1,334 genes) and 3,697 loss regions (250 genes). Among them, 10 candidate gene-copy losses and 18 candidate gene-copy gains were identified in the domestic goats compared with wild goats (Additional file 1: Table S18 and Table S19).

We randomly selected five genic CNVs from the candidate copy number gain genes. Then, we verified them in another four animals (one wild goat and three domesticated goats) using real-time quantitative PCR. All of the five genes show copy number variations (Additional file 1: Fig. S18). 80 % (4/5) of them show more copies in domesticated goats than the wild goat. Only one gene (NPAS3) shows more copies in the wild goat. Maybe NPAS3 is not a fixed gene duplication in domesticated goats, but show copy variation both in wild and domesticated individuals.

We also got the RNA-seq data from one wild goat brain tissue and one domestic brain tissue, based on our colleague's unpublished data. All of the candidate CNV genes in Additional file 1: Table 20 were investigated (Additional file 1: Table S23). 70.5 % (12/17) of the expressed gained genes show >2-fold increase expression in the Cashmere goat brain tissue, comparing with the wild goat brain tissue, which suggest that most of the genic CNVs could affect their gene expression level.

In the following sections, we detail a number of these CNV genes and rapidly evolving genes and explore their putative roles in the domestication and selection of domestic goats.

### Coat color evolution

Large coat-coloration variation is considered as one of the significant phenotypic characteristics in domestic animals compared with their wild ancestors [8]: wild ones often display uniform species-specific colors and patterns while domesticated ones possess a wide variety in both colors and patterns. Wild goats are generally tan-bodied with only subtle differences based on sex and age in contrast with domestic goats, which display a multitude of colors and patterns [34]. As for domestic goats, coat color is an important breed characteristic and production trait. For example, white coat color has reached the most common and dominant color in Angora goats as a result of strong artificial selection for white fibers. From the resequencing analysis, 13 genes mapped in CNV regions (*ASIP, ATRN,* Fig*.*4*, GNAQ, HELLS, MUTED, OSTM1, TRPM7, VPS33A, Adamts20, MITF, OCA2,* and *SLC7A11*) overlap with the cloned color gene list provided by European Society for Pigment Cell Research (http://www.espcr.org/micemut/) (Additional file 1: Table S21). The generation of the diversity in coat colors and patterns might be partially explained by the copy number changes of these coat color genes.

We assayed the agouti signaling protein gene (*ASIP*) in particular. Our analysis shows that *ASIP* is a single-copy gene in the tan wild goats (Fig. 2: Panel 1) as well as in the three black or brown full-bodied domestic breeds: the Yunnan Black goat (Fig. 2: Panel 2) and two Australian Rangeland goats (Fig. 2: Panel 3–4), but multi-copies were observed in other domestic goat breeds in the presence of white-color coat pattern (Fig. 2: Panel 5–7), among which white full-bodied Cashmere goat has the largest copy number. Basically, mammalian coat colors are determined by the relative amounts and distribution of two types of pigments: eumelanin (brown-black) and pheomelanin (yellow-red) [8]. Previous research indicates that ASIP acts on follicular melanocytes to inhibit α-MSH-induced eumelanin production in the melanogenesis pathway and hence generates tan-hair phenotype in wild-type form [35]. Here we not only confirm the role of copy number changes of *ASIP* gene in affecting coat color of domestic goat breeds shown in previous research [36, 37], but also reveal that *ASIP* gene duplication was widely used to contribute to the presence of domestic breeds with light coat color using whole-genome resequencing data, probably by decreasing the production of eumelanin through more *ASIP* copies.

According to color gene database, other 12 genes (*ATRN,* Fig*.*4*, GNAQ, HELLS, MUTED, OSTM1, TRPM7, VPS33A, Adamts20, MITF, OCA2* and *SLC7A11*) mapped in CNV regions are also associated with coloration. *ATRN, OSTM1* and *SLC7A11* are related with the synthesis of eumelanin and pheomelanin and the switch between them. Fig*.*4*, MUTED, VPS33A* and *OCA2* are involved in biogenesis of melanosome by controlling protein sorting and tracking in the process. *GNAQ, HELLS, Adamts20* and *MITF* occur in pigmentation during organ or tissue development. In particular, *MUTED* has fewer copies while *HELLS* and *OSTM1* gained more copies in all domestic goats than in wild goats (Additional file 1: Table S21). These genes might also play roles in coat color variation during goat domestication.

### Genes related to nervous system

Strong selection on the behaviors of less aggressive and reduced fear to human is often involved in animal domestication because tamer individuals, which were easier to handle, were preferred and thus preferentially retained by humans for breeding in the earliest stages of animal domestication. To better obtain insight into this adaptation, we investigated genes related with nervous system specifically. Among the 70 positively selected candidate genes (PSGs) we found (Additional file 1: Table S12 and S13), 20 genes (16 domestic goat genes and 4 wild goat genes) were found to function in nervous system associated processes (Additional file 1: Table S14), suggesting that positive selection of nervous systems-related genes may be a significant feature in goat or even all animal domestication. We were interested in whether these genes might underlie the molecular mechanism that led to the general behavior selection in initial domestication of animals.

The 5-hydroxytryptamine (5-HT) (or serotonin) concentration in central nerve system (CNS) is a major modulator of behavior in vertebrates and its reduced level in the serotonergic system is widely found to be strongly correlated with behavior disorder in human [38, 39]. Selective breeding for docility in the silver fox, over a period in excess of 50 years, found a marked increase of brain 5-HT [40], which is likely induced by the mutations or copy number variations of 5-HT pathway genes. Among PSGs we found that one PSG in domestic goat (*HTR3A*) and one PSG in wild goat (*CACNA1C*) are involved in 5-HT pathway. HTR3A (5-hydroxytryptamine receptor 3A) induces fast neural depolarizing after activation. *CACNA1C* is a voltage-gated ion channel gene and was found to be involved in serotonin release. Several genome-wide analysis researches have reported that *CACNA1C* is a top locus associated with schizophrenia and bipolar disorder [41, 42]. Rapid evolution of *CACNA1C* in wild goats may be driven by the need of alertness, while positive selection of *HTR3A* in domestic goats may be related to taming. The concrete functional significances of these 5-HT genes in behavior evolution during goat domestication await further functional molecular studies in the future.

Apart from 5-HT pathway analysis, the other 18 PSGs associated with nervous functions were found to take effect in regulating neuronal development and activity from different aspects, 15 of which were selected in domestic goats. Particularly, *CHD5*, *ULK1* and *TMEM132A* play roles in early brain development [43], neurogenesis [44] and neuronal differentiation [45, 46]. *NAV2* and *MGAT5B* function in neurite outgrowth and axon elongation [46, 47]. *SYNGR1* and *SYNDIG1* has essential functions in synaptic plasticity [48] and regulating excitatory synaptic strength [49].

We also found two other genes (*ERC2, GABRB2*) with nervous function duplicated in all domestic breeds based on CNV analysis, while they only have one copy in the two wild goats. In detail, ERC2 is involved in the neurotransmitter release at the nerve terminals active zone (CAZ) [50]. GABRB2 mediates inhibitory neurotransmission [51]. Our discovery on the behavior gene divergence between wild and domestic goats provides guiding clues on the selection of genes that should be investigated in the future studies understanding behavior evolution in domestic animals.

### Genes related to immune system and production traits

Compared with wild goats, domestic goats generally have more restricted environment imposed by human being thus different ranges of activity and food types. On the other hand, veterinary medicine has been widely practiced by human being since the ancient Egyptian times [52], which reflects human long-term care on their domesticated animals. In addition, it is well known that immune related genes usually evolve rapidly. Therefore, modulation on the immune system in domestication could be expected. In our analysis, we found four deleted gene copies (*ABCC4, PRAME, CD163L1,* and *KIR3DL1*) and two gained gene copies (*CFH* and *TRIM5*) in domestic goats involved in immune system. In detail, the multidrug transporter ABCC4 protects cells against toxicity by acting as anion efflux pump and also influences dendritic cell migration [53]. Analysis of *ABCC4* gene family also shows remarkable contraction in domestic goats (18 copies in wild goat but only 4 copies in domestic goats, which suggests that domestic goats may face less challenge from environmental biotoxins due to less wild food diversity and intensive human care. As for *CD163L1* gene, its expression could determine the response of γδ T cells to bacterial challenge [54]. Interestingly, a decrease of WC1^+^ γδ T cells in cattle occurred due to the absence of apparent infection when cattle were transferred from free-range grazing environment to conventional housing one with infertile food [55]. KIR3DL1 belongs to KIR (Killer-cell Ig-like receptors) family, which is highly diverse and rapidly evolved thus provides variability to the function of T-lymphocytes and NK cells and regulating immune responses to specific challenges [56]. As for gained genes in domestic goat, *CFH* (Complement factor H) is involved in the regulation of complement activation and thus the innate response against microbial infections [57]. TRIM5 is a retrovirus restriction factor, which mediates the innate immune defense against retroviruses infection [58]. In general, it is supported strongly that these genes are involved in the immune response against xenobiotics infections but expansion or contraction of individual gene families was likely driven by different types of pathogens specific to their own habitats, which help to trigger artificial selection for individuals with an adaptive immunity around human settlements.

**Fig. 3:**
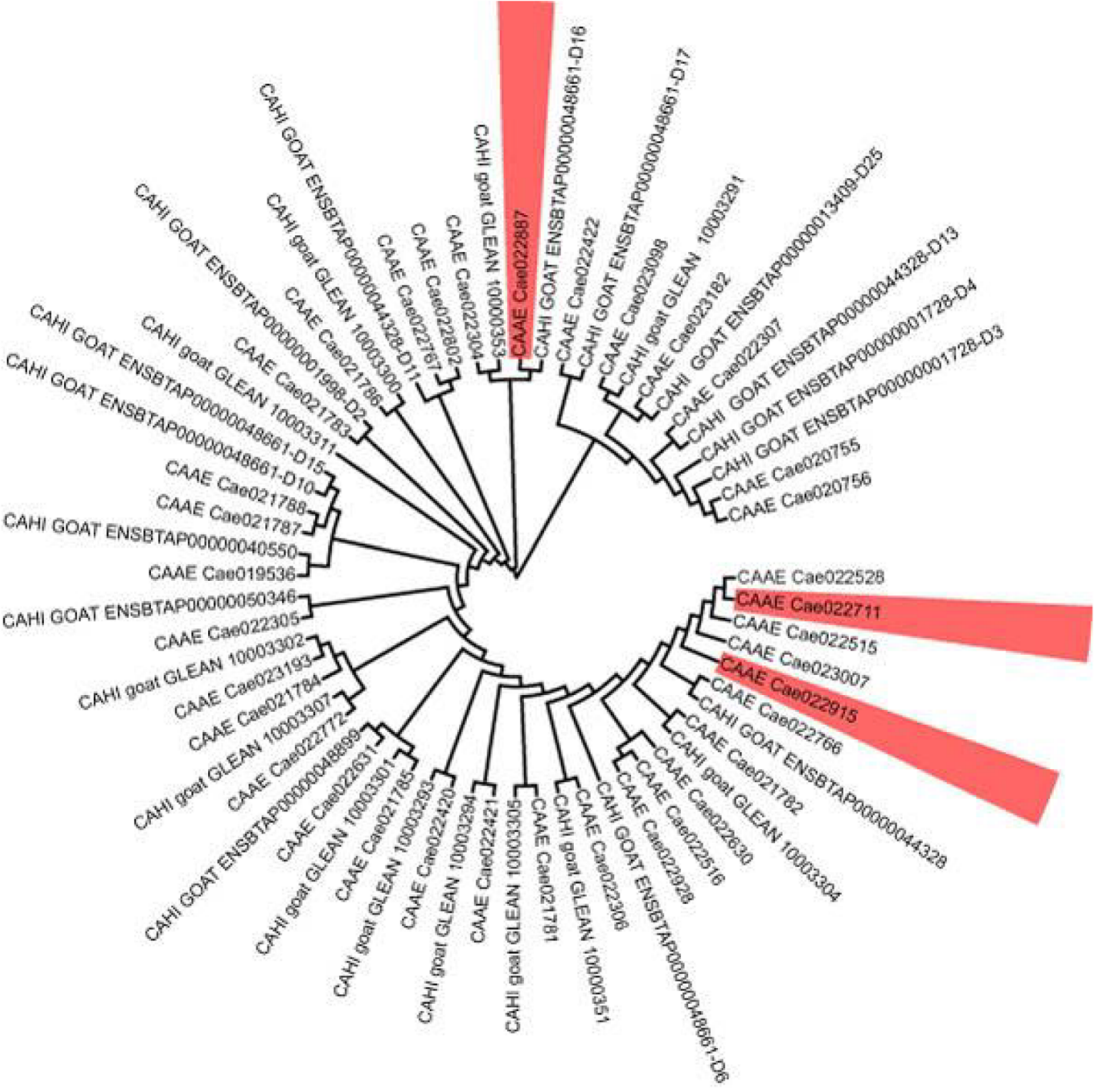
Molecular phylogenetic analysis of MYADM family by maximum likelihood method. (CAAE: gene in wild goat; CAHI: gene in domestic goat) Three copies in wild goat MYADM family highlighted with red, Cae022711, Cae022915 and Cae022887, were detected as losses in all domestic goat breeds

We also found three production-trait related genes from CNV analysis which show copy losses in all our sequenced domestic goats: *MYADM*, *BTN1A1* and *PRAME*. BTN1A1 is a major protein associated with lipid droplets in the milk and found to be essential for secretion of milk-lipid droplets [59]. *PRAME* gene family is reportedly involved in adaptive functions including spermatogenesis and immunity [60]. In particular, *MYADM* has been found to be highly associated with erythrocyte morphology and weight of weaned lamb [61]. Phylogenetic analysis on the 36 *MYADM* copies in wild goat and 27 ones in domestic goat (Fig. 3) were used to determine the closest common ancestor of *MYADM* gene copies between two lineages and found a contraction of the of *MYADM* gene family in domestic goats. These gene families might be important targets in strong human selection of individuals with high production traits in breeding.

Moreover, four PSGs involved in the lipid metabolism pathway (wild goat: *ACSL1*; domestic goat: *LRP1*, *PLIN4* and *FASN*) were identified. Specially, *ACSL1* (Long-chain-fatty-acid--CoA ligase 1) functions in fatty acid degradation while *FASN* (Fatty acid synthase) functions in fatty acid synthesis and LRP1 (Low density lipoprotein receptor-related protein 1) stimulates fatty acid synthesis. *FASN* has been shown to affect bovine adipose fat composition and milk fat composition [62]. These modification in fatty acid metabolism-related genes would likely function on domestic goat body fat and milk fat composition [63].

**Fig. 4:**
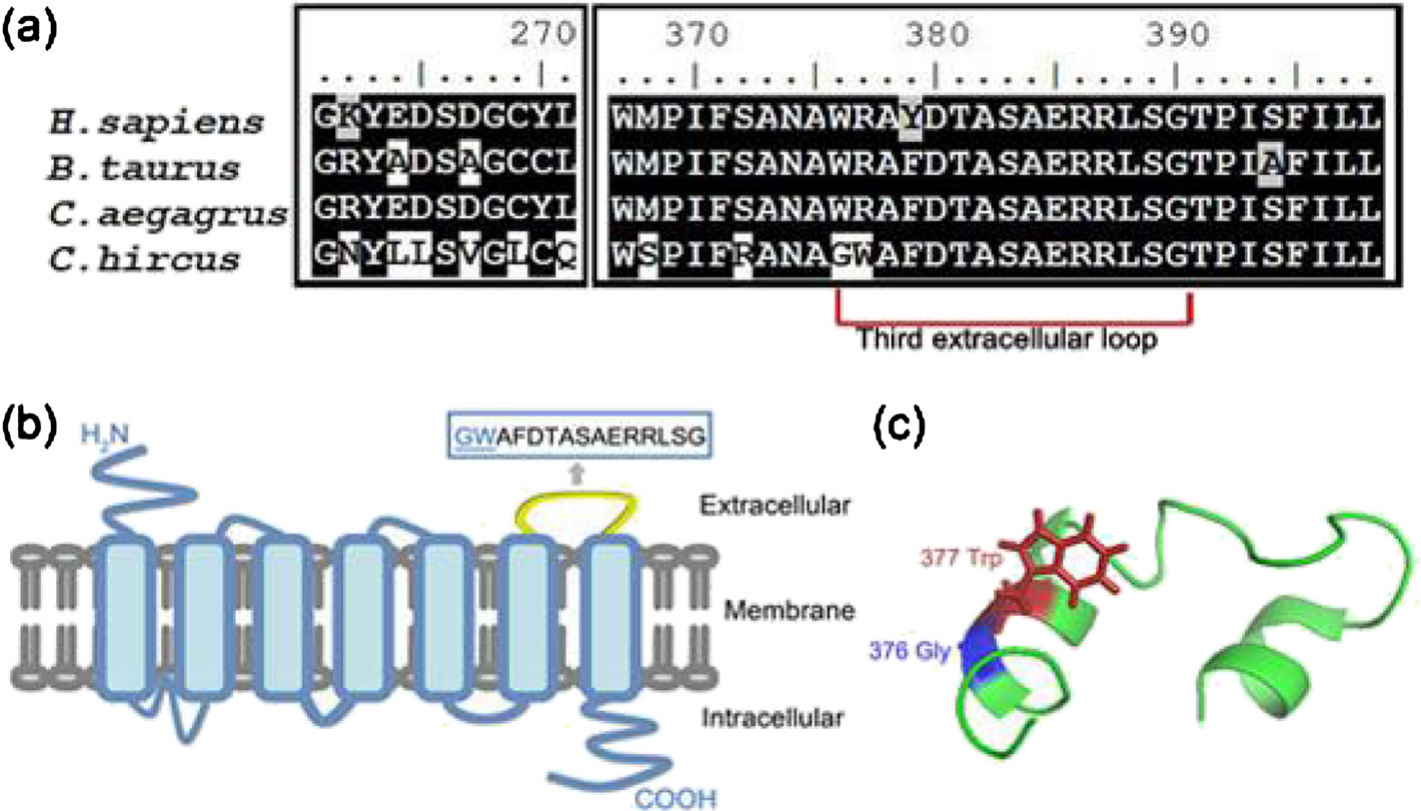
Unique amino acid changes in CCKA receptor (CCKAR) sequences and their roles in satiety regulation in domestic goats. (**a**) Alignment of CCKA receptor sequences among human (*H. sapiens*), cattle (*B. taurus*), wild goat (*C. aegagrus*) and dometic goat (*C. hircus*). Amino acids unique to domestic goats are shown. Completely identical residues in all receptor homologues are shown in white letters with black background, similar residues are shown in black letters with gray background, and distinct residues are shown in black letters with white background. (**b**) Transmembrane topology of CCKAR. The binding region (the third extracellular loop) affected in the domestic goat is highlighted with yellow. (**c**) Structural model of the third extracellular loop of CCKAR. Locations of the ligand binding loop with altered sequences in the domestic goat are shown

It is intriguing that a PSG *CCKAR* (*Cholecystokinin A receptor*) in domestic goat is a satiety signaling receptor gene. Previous research shows that this gene is a strong candidate for growth characteristics from chicken QTL mapping experiments, whose expression level is decreased in high-growth birds and induce appetite promoting [64]. *CCKAR* of domestic goat changed uniquely at amino acids Asn 262, Leu 264, Leu 265, Val 267, Leu 269, Gln 271, Ser 368, Arg 372, Gly 376 and Trp 377 (Leu 264, Leu 265 and Leu 269 are calculated as positive selected, *P* < 0.05, likelihood ratio test for the branch-site model), and the latter two residues located in the conserved ligand (Cholecystokinin-8, CCK-8) binding motif (the third extracellular loop) of CCKAR [65] (Fig. 4). The altered residue Glycine at site 376 changed the previous Trp-Trp interaction to Trp-Gly interaction, and thus it may disorder the alpha helix in the loop [66] and lose the tight association with Trp 30 of ligand CCK-8 side chain. What’s more, the mutated Trp 377 should replace the former strong salt bond between positively charged CCKAR Arg 377 and ligand CCK-8 side chain of negatively charged Asp 32 with weaker Trp-Asp electrostatic interaction [67]. Taking together, the mutated amino residues would likely decrease the binding affinity of CCKAR/CCK-8 around the third extracellular loop of CCKAR and thus partially inhibit the effect of CCK and result in decreasing satiety and promoting appetite in domestic goats. This finding might also strengthen the hypothesis that domestication process may involve the selection for individual differences in appetite in mammals, which results in individual differences in growth rate [68]. We also notice that the *IGF1R* (*insulin-like growth factor 1 receptor*), which plays an important role in growth and contribute to dog body size in domestication with specific mutation [69] was positively selected in wild goats. But it is unknown why this gene was selected in the wild goat.

## Conclusions

Our comparative and evolutionary analyses based on comparison between wild and domestic goat reference genomes and resequencing of different goat breeds have provided important insights into the evolution and domestication of domestic goats. By CNV and rapidly evolving genes studies, we identified genes associated with coat color evolution, behavior traits, immune response and production traits, which are important targets in goat domestication. The genes with domestic goat-specific variations identified here not only provide guidance gene list for further functional characterization on domestication genes in goats, they will also lend useful information for understanding genetic mechanism during animal domestication in general. These genes will also be useful markers in future goat breeding. This study highlights the value of comparing reference genomes between domestic animals and their wild progenitors combined with resequencing data in mining artificially selected candidate genes during animal domestication.

## Additional files

Additional file 1: **Contains Supplementary** **Figures S1–S13** **and** **Tables S1–S23** **and Supplementary methods.**

Additional file 2: **Contains 70 sequence alignment files respectively for the 70 PSGs; and further supporting data is available from the authors on request.**
