# Supplementary material for: Reference genome of wild goat (capra aegagrus) and sequencing of goat breeds provide insight into genic basis of goat domestication
Source: BMC Genomics. 2015 Jun 5;16(1):431. doi: 10.1186/s12864-015-1606-1 (PMC4455334; doi:10.1186/s12864-015-1606-1)
Supplement: Supplementary file 1 — Contains Supplementary Figures S1–S13 and Tables S1–S23 and Supplementary methods. [file 12864_2015_1606_MOESM1_ESM.pdf]

Additional file1

1 Supplementary Figures.....3
1.1 Supplementary Fig. S1. Geographical distribution of wild goat samples gathered.....3
1.2 Supplementary Fig. S2. Distribution of 17-mer frequency in the sequencing reads.....4
1.3 Supplementary Fig. S3. Distribution of sequencing depth of the assembled wild goat genome.....5
1.4 Supplementary Fig. S4. G+C content distributions for wild goat and relative species genome.....6
1.5 Supplementary Fig. S5. TE annotation assessment.....7
1.6 Supplementary Fig. S6. Comparison of gene features among the sequenced related species......8
1.7 Supplementary Fig. S7. Comparison of protein orthology among genomes of eight mammalians.....9
1.8 Supplementary Fig. S8. Venn diagram showing the number of unique and shared gene families among wild goat, domestic goat, sheep and other five species：cattle, pig, horse, dog and human......10
1.9 Supplementary Fig. S9. Estimation of the time of divergence.....11
1.10 Supplementary Fig. S10. The dynamic evolution of orthologous gene families..12
1.11 Supplementary Fig. S11. GO enrichment analysis of the genes located in CNV regions.....13
1.12 Supplementary Fig. S12. Distribution of orthologous protein similarity between wild goat and domestic goat and between wild goat and sheep.....14
1.13 Supplementary Fig. S13. Validation of CNVs using qPCR.....15

1.14 **2 Supplementary Tables**.....16
1.15 Supplementary Table S1. Clones and reads used in the sequencing of the wild goat genome.....16
1.16 Supplementary Table S2. Statistics of 17-mer analysis.....17
1.17 Supplementary Table S3. Summary of assembled genome of wild goat.....18
1.18 Supplementary Table S4. Comparative statistics of the genome assembly of wild goat and domestic goat.....19
1.19 Supplementary Table S5. Statistics of the completeness of the wild goat genome based on 248 CEGs.....20
1.20 Supplementary Table S6. Comparison of repeat sequence between wild goat and its related species.....21
1.21 Supplementary Table S7. Non-coding RNA genes in the Wild goat genome.....22
1.22 Supplementary Table S8. General Statistics of Predicted Protein-coding Genes.23
1.23 Supplementary Table S9. Number of Genes with Homology or Functional Classification by each Method.....24
1.24 Supplementary Table S10. Statistics of gene family clustering.....25

1.25 Supplementary Table S11. Statistics of dynamic change of olfactory receptor gene family....26
1.26 Supplementary Table S12. Positive selected genes (PSGs) in wild goat....27
1.27 Supplementary Table S13. Positive selected genes (PSGs) in domestic goat....27
1.28 Supplementary Table S14. Positive selected genes associated with nervous system....30
1.29 Supplementary Table S15. Statistics of pseudo-chromosome assembly....31
1.30 Supplementary Table S16. Genes in the wild goat Y psedu-chromosome....32
1.31 Supplementary Table S17. Information about five re-sequencing Australian goat sample....35
1.32 Supplementary Table S18. Highly confident deleted gene copies in domestic goats....36
1.33 Supplementary Table S19. Highly confident gained gene copies in domestic goats....37
1.34 Supplementary Table S20. Classification of representative copy gain and loss genes in domestic goat based on functions....38
1.35 Supplementary Table S21. Copy number variations of color genes among wild goat and domestic goat breeds....39
1.36 Supplementary Table S22. qPCR primers used for the verification of five picked candidate gained gene copies in domestic goats....40
1.37 Supplementary Table S23. The comparison of gene expression level between one wild goat brain tissue and one Cashmere goat brain tissue, for the candidate gain and loss genes in domestic goats....41

1.38

1.39 **3 Supplementary Methods**....43
1.40 Data Generation....43
3.1.1 Sequencing....43
3.1.2 Filtering raw data....43
3.1.3 Kmer Analysis....44
3.1.4 Genome assembly....45
3.1.5 Evaluation of the assembled genome sequence....46
3.1.6 Anchoring scaffolds to chromosomes....47
3.1.7 Repeat annotation....48
3.1.8 Gene annotation....49
3.1.9 Gene function annotation....51
3.1.10 ncRNA annotation....51
1.41 Analysis of gene families....50
3.2.1 Identification of wild goat gene families....51
3.2.2 Construction of phylogenetic tree....51
3.2.3 Expansion and contraction of gene families....51
1.42 Rapidly evolving gene analysis....53

1.43

1.44 **4 Supplementary Reference**....55

1 Supplementary Figures

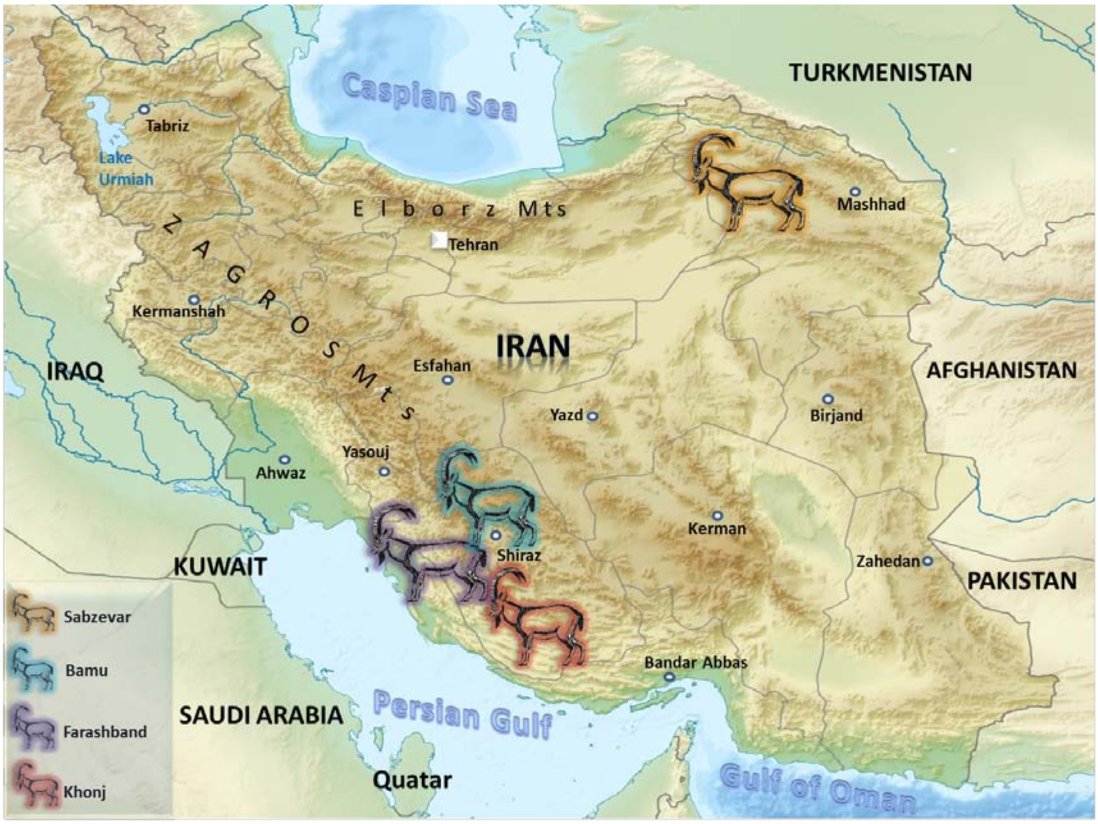

Supplementary Fig. S1. Geographical distribution of wild goat samples gathered

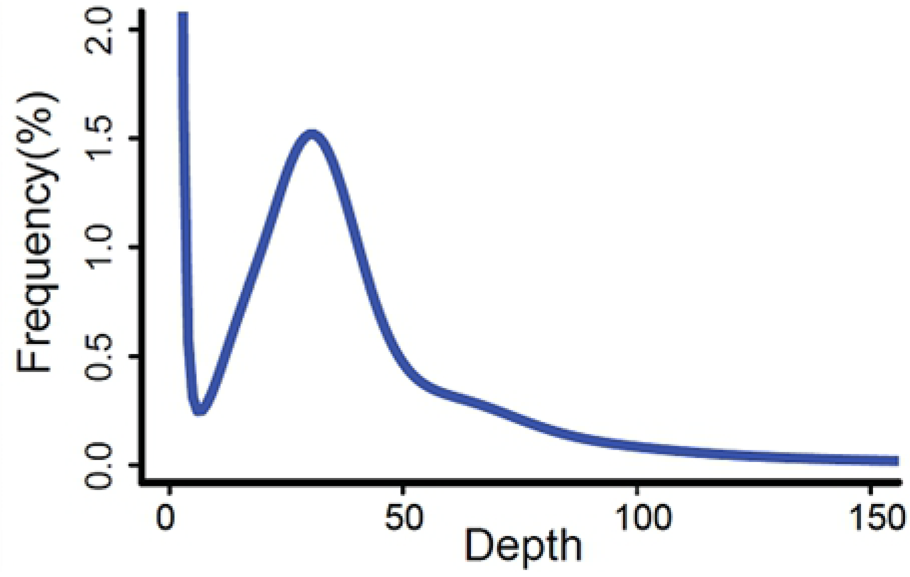

Supplementary Fig. S2. Distribution of 17-mer frequency in the sequencing reads. The peak of the 17-mer distribution is about 30 X and then the genome size can be estimated as 2.90 Gb.) (See Supplementary Method 3.1.3)

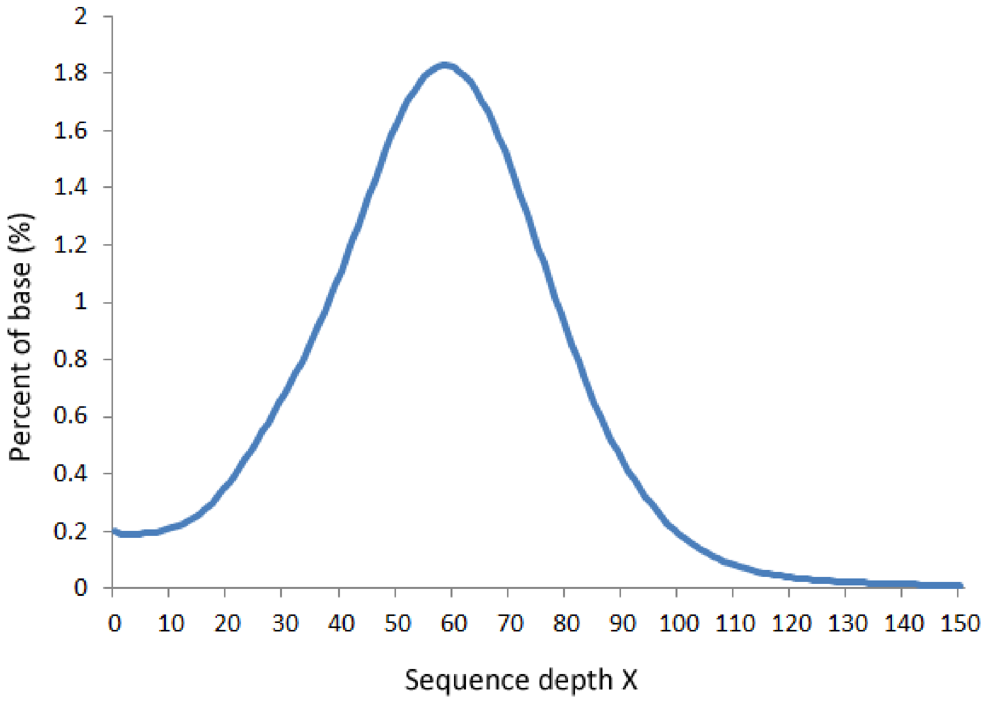

Supplementary Fig. S3. Distribution of sequencing depth of the assembled wild goat genome. The x-axis is the single-base sequencing depth and the y-axis is the proportion of the base number divided by the total bases in assembled genome sequence.

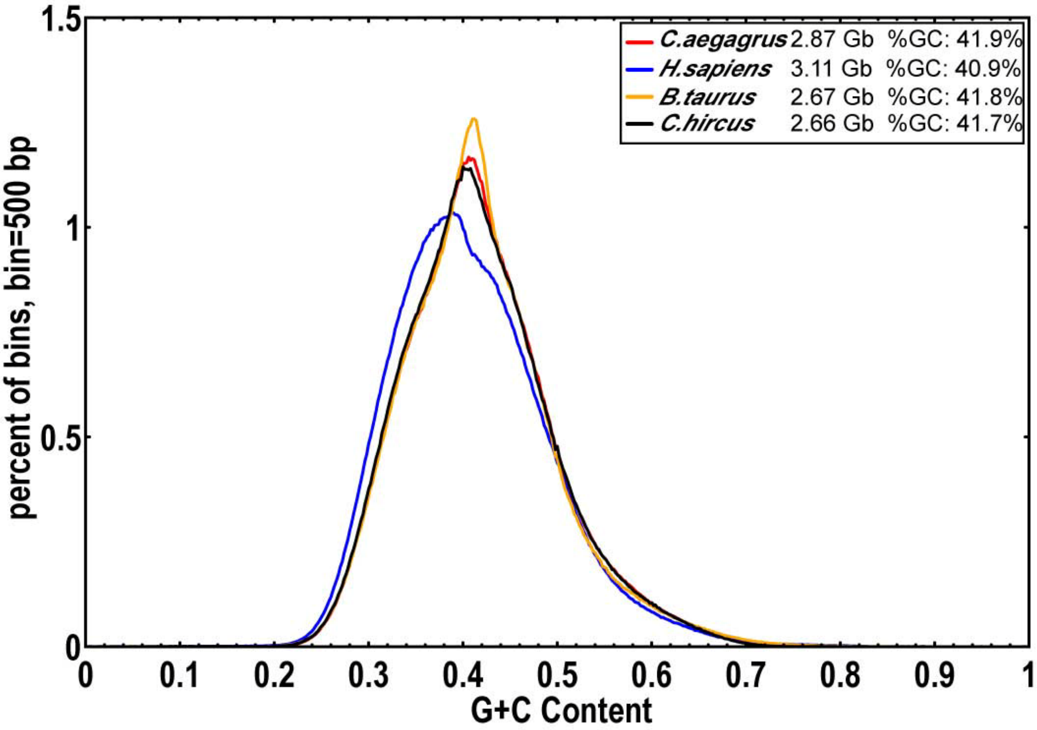

Supplementary Fig. S4. G+C content distributions for wild goat and relative species genome. The x-axis is GC content and the y-axis is the proportion of 500 bp non-overlapping sliding windows with a given GC content. (See Supplementary Method 3.1.5)

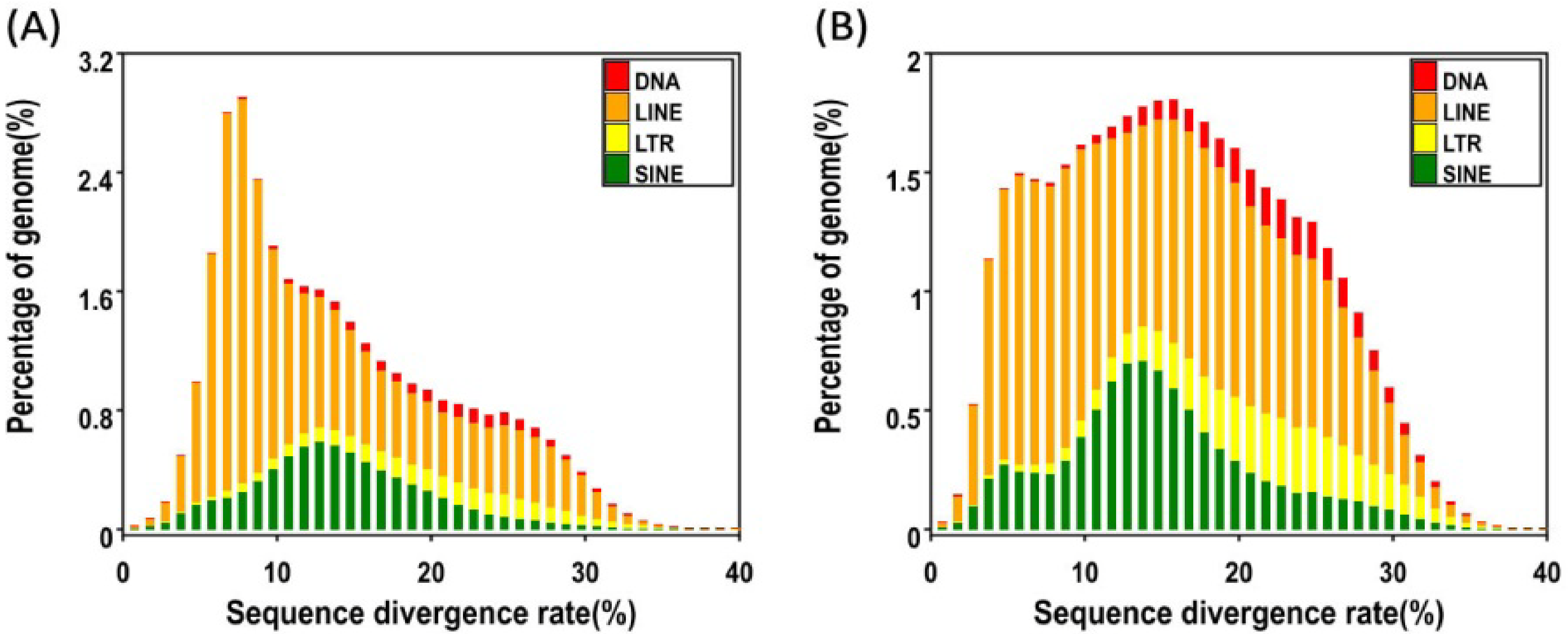

Supplementary Fig. S5. TE annotation assessment: distribution of divergence rate of each type of wild goat's TE

(A) The divergence rate between the identified TE elements in the genome by *de novo* method and the consensus sequence deposited in the predicted TE library was calculated.
(B) The divergence rate between the identified TE elements in the genome by homology-based method and the consensus sequence deposited in the Repbase library was calculated.

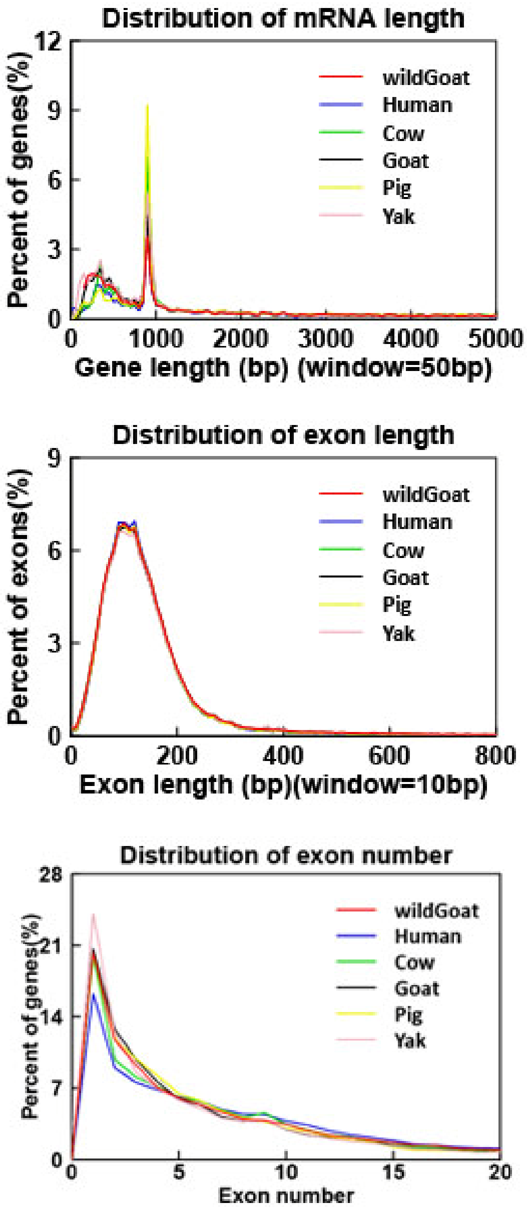

Supplementary Fig. S6. Comparison of gene features among the sequenced related species.

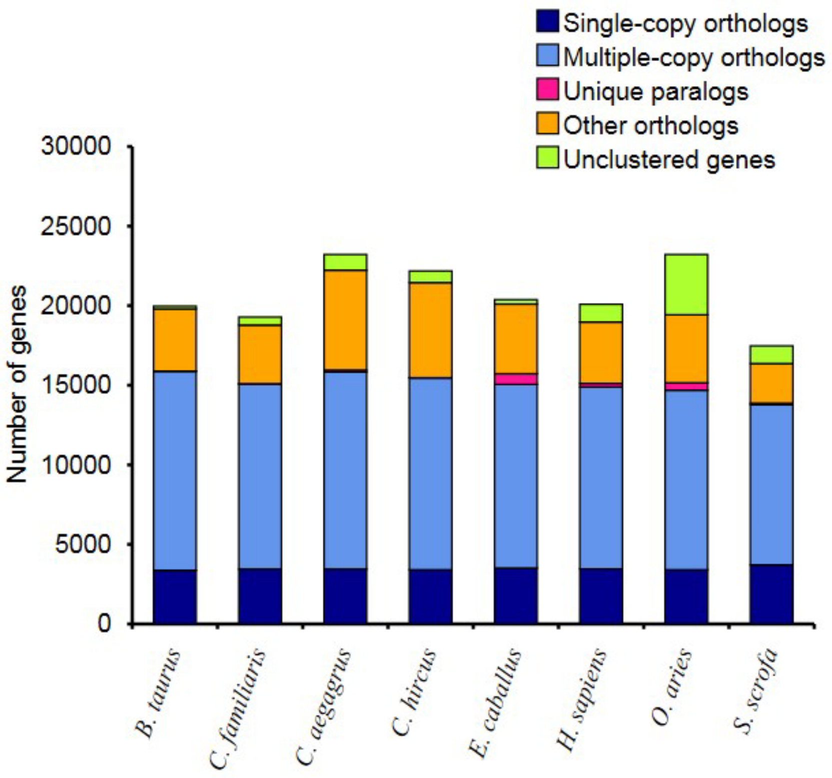

Supplementary Fig. S7. Comparison of protein orthology among genomes of eight mammalians: wild goat, domestic goat, sheep, cattle, pig, horse, dog and human (*C.aegagrus*, *C.hircus*, *O.aries*, *B.taurus*, *S.scrofa*, *E.caballus*, *C.familiaris* and *H.sapiens*)

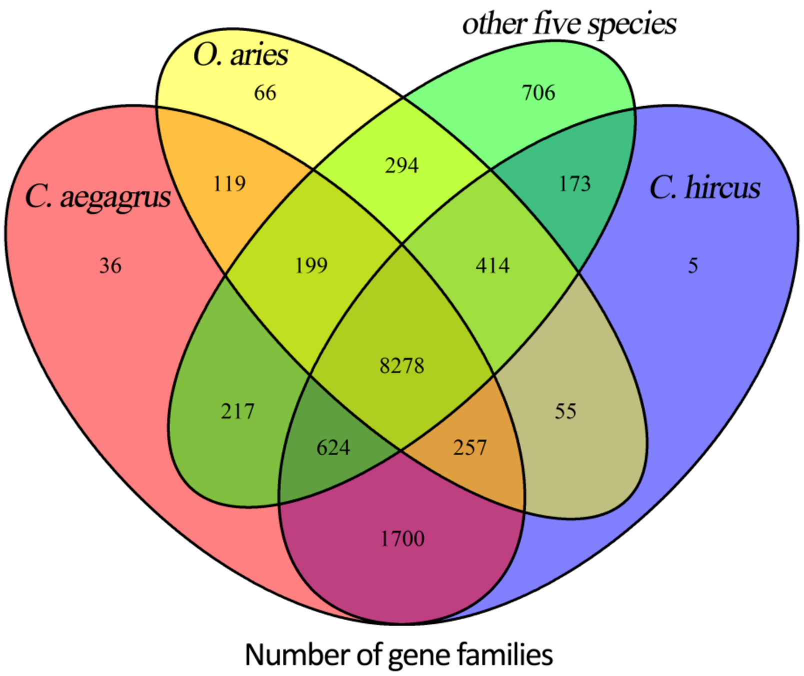

Supplementary Fig. S8. Venn diagram showing the number of unique and shared gene families among wild goat, domestic goat, sheep and other five species: cattle, pig, horse, dog and human.

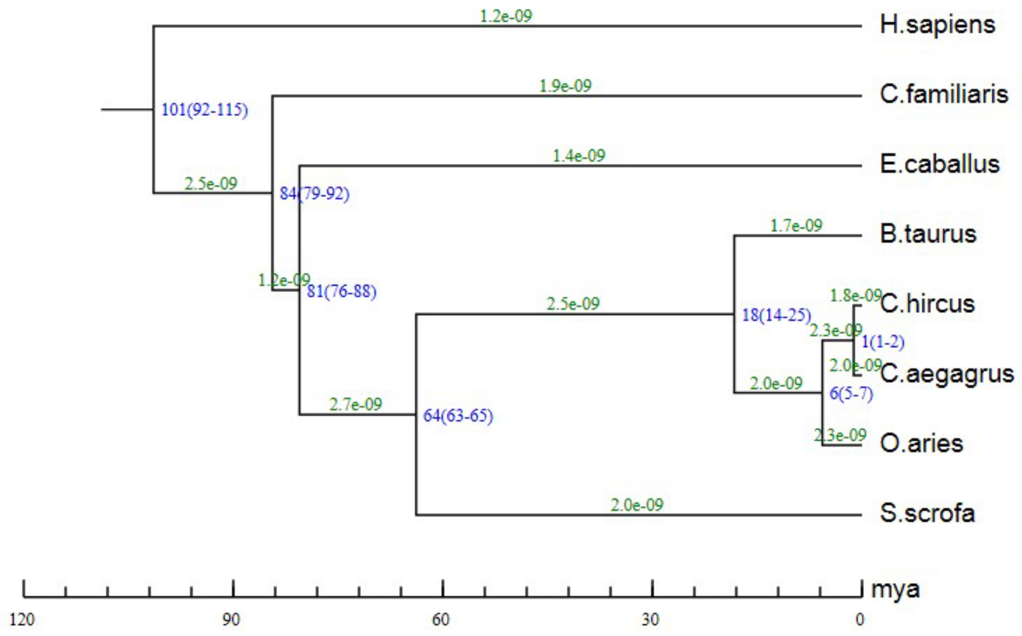

Supplementary Fig. S9. Estimation of the time of divergence. Estimation of the time of divergence (with error range shown in parentheses) of the wild goat and seven other mammals based on orthology relationship. Distances are shown in years with green color: the divergence time between wild goat and domestic goat is estimated about 1 million year. This phylogenetic tree have been submitted to TreeBase (http://purl.org/phylo/treebase/phylows/study/TB2:S17499).

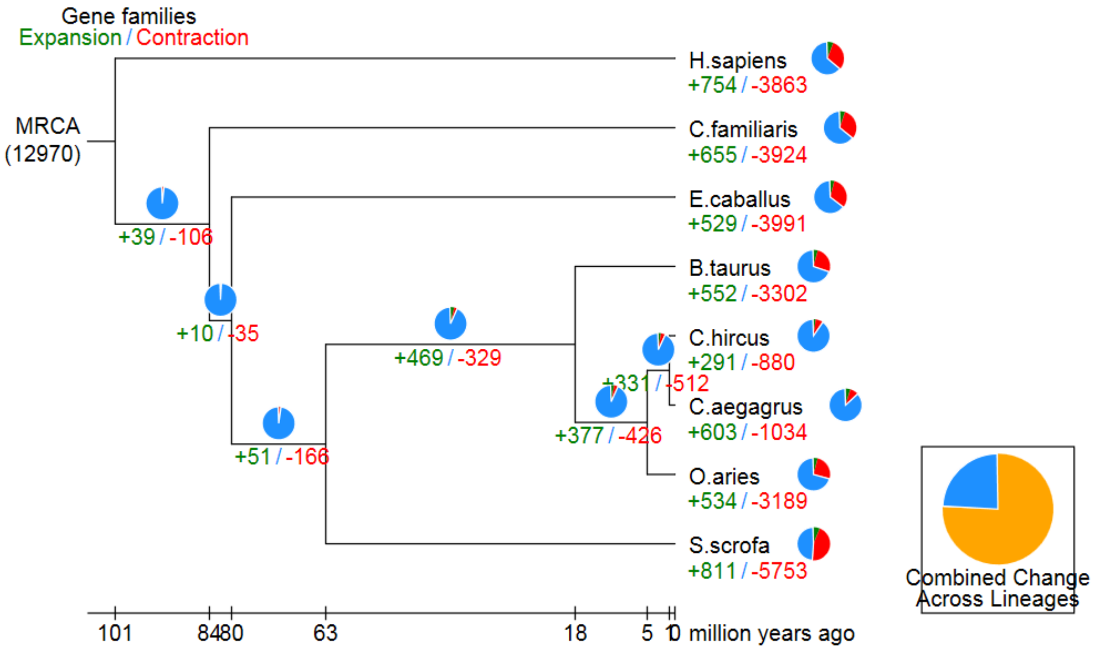

Supplementary Fig. S10. The dynamic evolution of orthologous gene families.

MRCA refers to “most recent common ancestor”.

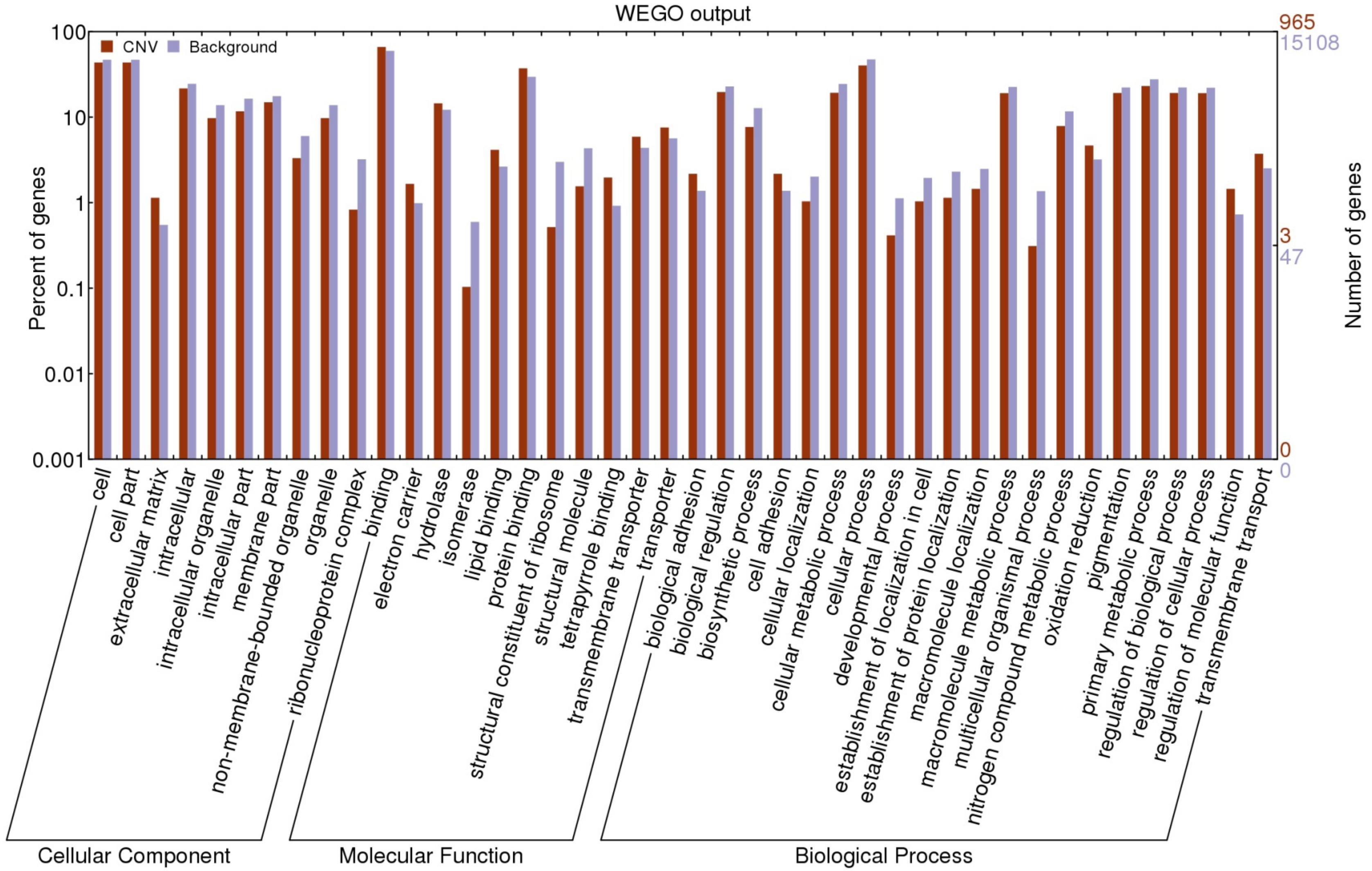

Supplementary Fig. S11. GO enrichment analysis of the genes located in CNV regions. This analysis was completed by WEGO[1] (only show terms with P <0.05, $\boldsymbol{x^2-\text{test}}$).

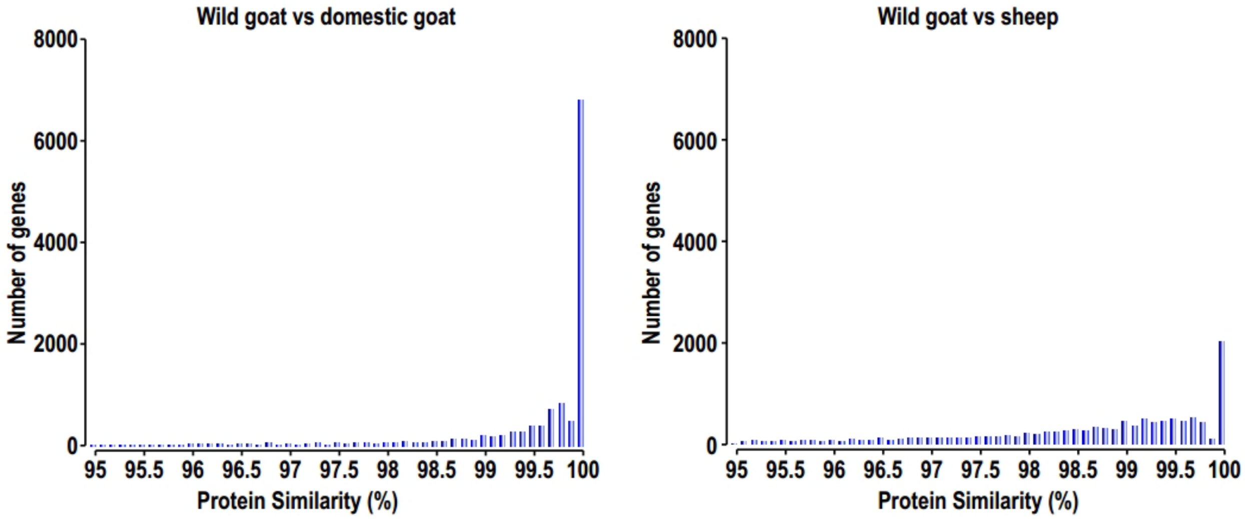

Supplementary Fig. S12. Distribution of orthologous protein similarity between wild goat and domestic goat and between wild goat and sheep.

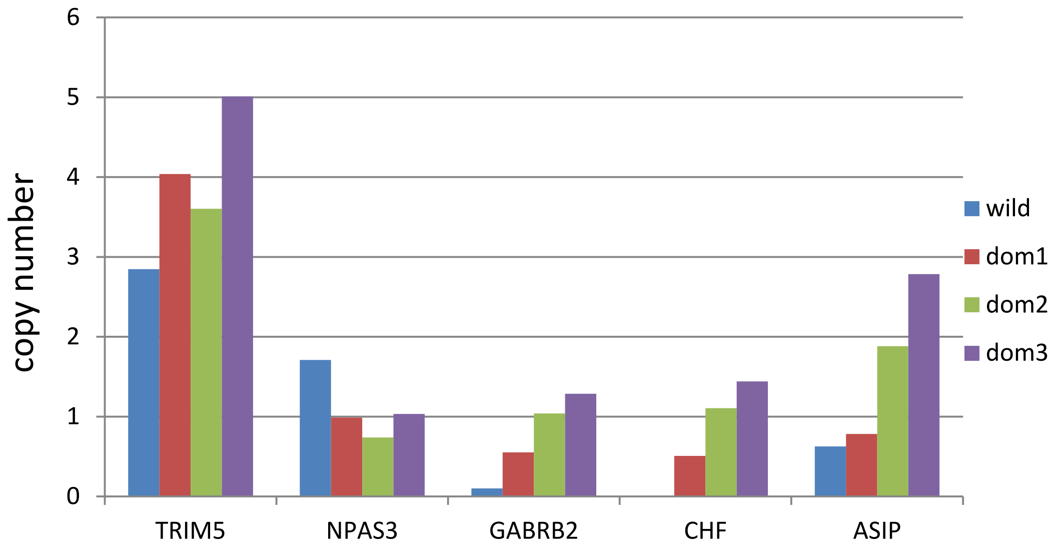

Supplementary Fig. S13. Validation of CNVs using qPCR.

Histogram showing the copy number in one wild goat (wild), one Yunnan Black goat(dom1) and two Inner Mongolian white Cashmere goats (dom2 and dom3), as the single copy gene, C7orf28b, is control gene.

2 Supplementary Tables

Supplementary Table S1. Clones and reads used in the sequencing of the wild goat genome

Wild goat sample source	Paired-end insert size	#Library	Raw reads				Filtered reads			
			Reads Length (bp)	Total Data (Gb)	Sequence coverage (X)	Physical coverage (X)	Reads Length (bp)	Total Data (Gb)	Sequence coverage (X)	Physical coverage (X)
Bamu	250bp	1	150/150	59.94	21.41	17.84	130/130	43.13	15.4	14.81
Bamu	500bp	1	150/150	48.88	17.46	29.1	130/125	35.76	12.77	25.04
Bamu	800bp	4	150/150	79.98	28.58	76.18	130/125<br>95/95<br>95/90	49.86	17.8	67.13
Bamu	2000bp	3	49/49	53.67	19.16	391.16	49/49	40.05	14.31	291.94
Bamu	5000bp	3	49/49	61.43	21.93	1119.2	49/49	35.82	12.8	652.71
Khonj	10000bp	2	49/49	38	13.57	1384.65	49/49	16.85	6.02	614
Farashband, Sabzevar	20000bp	2	49/49	39.6	14.15	2886.52	49/49	12.43	4.44	905.93
	Total	16	-	381.5	136.26	5904.65	-	233.90	83.54	2571.56

Supplementary Table S2. Statistics of 17-mer analysis

Kmer	#Used read	#Used base	Sequencing coverage	Kmer_num	Peak_ depth	Genome size(bp)
17	773,730,862	99,479,919,110	34	87,100,225,318	30	2,903,340,843

Supplementary Table S3. Summary of assembled genome of wild goat

	Contig[a]		Scaffold	
	**Size(bp)**	**Number**	**Size(bp)**	**Number**
N90	4,048	150,295	385,908	1,544
N80	7,815	104,724	809,824	1,046
N70	11,324	76,848	1,227,514	761
N60	14,997	56,624	1,613,611	557
N50[b]	18,965	40,979	2,057,686	399
Longest	209,451	-	11,374,952	-
Total Size[c]	2,639,298,746	-	2,873,293,603	-
Total Number (>=100b)	-	727,076	-	454,028
Total Number (>=2kb)	-	187,572	-	7,499
Gap ratio	-	0.00%	-	8.15%

a. The contig refers to the final contig after filling the gap of intra-scaffold.
b. N50 size is a weighted median statistic indicating that 50% of the entire assembly resides in contigs/scaffolds of a length at least X. N60, N70, N80 and N90 are similarly defined.
c. The length shorter than 100bp was not included in the statistics.

Supplementary Table S4. Comparative statistics of the genome assembly of wild goat and domestic goat

	Wild goat	Domestic goat
Coverage (X)	83.54	65.6
Contig N50 (bp)	18,965	18,720
Total length of contigs (Gb)	2.64	2.52
Scaffold N50 (bp)	2,057,686	2,212,139
Total length of scaffolds (Gb)	2.87	2.66
Repeat (bp)	1,415,433,349	1,280,884,817
Protein coding gene (bp)	740,060,950	664,536,174
ncRNA (bp)	5,382,317	398,934
Total annotated sequence (bp)	2,160,876,616	1,945,819,925

Supplementary Table S5. Statistics of the completeness of the wild goat genome based on 248 CEGs[a]

	CEGs number	mapped Proteins	%Completeness	Total	Paralogy index[c]
**Complete[b]**	**248**	**243(242)[d]**	**97.98(97.58)**	**393(387)**	**35.39(35.12)**
Group 1	66	63	95.45	90	26.98
Group 2	56	55	98.21	75	23.64
Group 3	61	60	98.36	94	33.33
Group 4	65	65	100	134	55.38
**Partial[b]**	**248**	**246(247)**	**99.19(99.6)**	**417(415)**	**39.02(38.46)**
Group 1	66	64	96.97	94	29.69
Group 2	56	56	100	77	25
Group 3	61	61	100	102	37.7
Group 4	65	65	100	144	61.54

a. The CEGs database contains groups of genes from the following species: *Homo sapiens*, *Drosophila melanogaster*, *Arabidopsis thaliana*, *Caenorhabditis elegans*, *Saccharomyces cerevisiae* and *Schizosaccharomyces pombe*. The CEGs were classed as 4 groups based on the conservation.
b. Complete and partial refer to the length of alignments.
c. Paralogy index is the proportion of mapped CEGs within paralogs.
d. Evaluation data of domestic goat genome were showed in the parentheses.

Supplementary Table S6. Comparison of repeat sequence between wild goat and its related species

	Wild goat		Domestic goat		Sheep		Cattle	
	Length(bp)	Coverage (%)	Length(bp)	Coverage (%)	Length(bp)	Coverage (%)	Length(bp)	Coverage (%)
**Tandem repeat**	57,001,424	2.16	37,108,135	1.39			66,275,669	2.27
**LINE:**								
L1	338,555,508	11.78	349,201,302	13.11	319,721,973	12.14	328,664,804	11.26
RTE (BovB)	384,520,262	13.38	292,087,967	10.97	359,281,823	13.65	313,409,818	10.74
L2	28,832,188	1.00	28,595,723	1.07	26,886,976	1.02	34,553,185	1.18
CR1	2,822,812	0.10	2,618,832	0.10	2,069,573	0.08	3,083,954	0.11
other	6,200,393	0.22	4,353,483	0.16	20,895,507	0.79	NA	NA
Total	760,931,163	26.48	676,857,307	25.42	728,855,852	27.68	679,711,761	23.29
**SINEs:**								
BOV-A	105,719,828	3.68	109,301,376	4.10	86,706,307	3.29	68,880,046	2.36
Bov-tA	NA	NA	NA	NA	NA	NA	225,579,571	7.73
ART2A	NA	NA	NA	NA	NA	NA	121,997,595	4.18
tRNA	76,598,813	2.67	87,328,631	3.28	60,289,254	2.29	57,981,206	1.99
MIR	30,816,619	1.07	31,738,801	1.19	29,844,110	1.13	40,569,445	1.39
Other	904,913	0.03	1,142,465	0.04	735,261	0.03	432,334	0.01
Total	214,040,173	7.45	229,511,273	8.62	177,574,932	6.74	515,440,197	17.66
**LTR:**								
ERVs	132,209,363	4.60	123,727,202	4.65	121,689,503	4.62	93,363,384	3.20
LTR other	4,238,914	0.15	2,557,812	0.10	2,709,230	0.10	12,395,410	0.42
Total	136,448,277	4.75	126,285,014	4.74	124,398,733	4.73	105,758,794	3.62
**DNA transposon**	62,389,525	2.17	60,428,396	2.27	59,832,665	2.27	57,157,641	1.96
**Other**	13,730,376	0.48	12,903,482	0.48	26,753,185	1.02	NA	NA
**Interspersed repeat total**	1,187,539,514	41.33	1,105,985,472	41.54	1,117,415,367	42.44	1,358,068,393	46.54

Supplementary Table S7. Non-coding RNA genes in the Wild goat genome

Type		Copy	Average length(bp)	Total length(bp)	% of genome
miRNA		433	89.75	38862	0.001353
tRNA		685	75.02	51391	0.001789
rRNA	rRNA	491	113.61	55784	0.001941
	18S	60	150.60	9036	0.000314
	28S	165	151.15	24940	0.000868
	5.8S	7	116.43	815	0.000028
	5S	259	81.05	20993	0.000731
snRNA	snRNA	973	113.74	110673	0.003852
	CD-box	279	91.85	25626	0.000892
	HACA-box	225	135.68	30527	0.001062
	splicing	443	115.97	51376	0.001788

Supplementary Table S8. General Statistics of Predicted Protein-coding Genes

Gene Set		Number	Average Transcript Length(bp)	Average CDS Length (bp)	Average Exons per Gene	Average Exon Length (bp)	Average Intron Length (bp)
Homolog	*H.sapiens*	19,342	41,602.50	1,538.09	8.95	171.77	5,036.83
	B.taurus	23,811	29,792.91	1,397.41	7.95	175.82	4,087.00
	C.hircus	26,266	27,212.08	1,254.14	7.20	174.12	4,184.84
	B.grunniens	28,367	32,719.15	1,254.66	6.99	179.48	5,252.32
	S.scrofa	23,942	23,379.15	1,235.74	6.91	178.88	3,747.81
Merger 1		20,116	35,212.22	1,405.22	8.33	168.67	4,611.24
De novo	AUGUSTUS	20,195	50,391.37	1,435.49	8.73	164.41	6,332.15
	Genescan	41,384	43,021.42	1,240.28	7.69	161.35	6,248.13
	GlimmerHMM	19,453	9,122.59	861.96	5.25	164.05	1,941.71
EST	*C.hircus*	36,611	190,585.64	416.33	2.57	162.11	121,273.10
	O.aries	413,644	231,894.44	519.50	2.87	180.87	123,585.60
Transcriptome	*C.hircus*	8,300	28,427.49	1,948.13	11.81	164.98	2,449.88
Merger 2		470	39,034.25	1,203.26	6.84	175.90	6,477.44
Glean		15,192	9,407.60	1,111.78	5.84	190.21	1,712.27
Final gene set		23,217	31,876.82	1,330.69	7.74	171.89	4,530.90

Supplementary Table S9. Number of Genes with Homology or Functional Classification by each Method

		Number	Percentage (%)
Annotated	Swissprot	20,881	89.94
	TrEMBL	21,314	91.80
	KEGG	16,140	69.52
	InterPro	17,993	77.50
	GO	15,108	65.07
Unannotated		1859	8.01
Annotated		21,358	91.99

Supplementary Table S10. Statistics of gene family clustering

Species	Genes Number	Genes in Families	Unclustered Genes	Family Number	Unique Families	Average Genes per Family
B.taurus	*19,970*	*19,804*	*166*	*9,488*	*2*	*2.09*
C.familiaris	*19,281*	*18,781*	*500*	*9,403*	*11*	*2*
C.aegagrus	*23,217*	*22,234*	*983*	*11,430*	*36*	*1.95*
C.hircus	*22,175*	*21,452*	*723*	*11,506*	*5*	*1.86*
E.caballus	*20,383*	*20,103*	*280*	*9,321*	*19*	*2.16*
H.sapiens	*20,087*	*18,973*	*1,114*	*9,563*	*63*	*1.98*
O.aries	*23,220*	*19,426*	*3,794*	*9,682*	*66*	*2.01*
S.scrofa	*17,480*	*16,364*	*1,116*	*8,322*	*44*	*1.97*

Supplementary Table S11. Statistics of dynamic change of olfactory receptor gene family

	Gene No.(Wild goat)	Gene No. (Domestic goat)
Total	1134	930
Clustered	1093	893
Unclustered	41	37

Supplementary Table S12. Positive selected genes (PSGs) in wild goat

Gene	Annotation
Cae000752	SSH1_HUMAN Protein phosphatase Slingshot homolog 1 OS=Homo sapiens GN=SSH1 PE=1 SV=2
Cae001820	ACSL1_HUMAN Long-chain-fatty-acid--CoA ligase 1 OS=Homo sapiens GN=ACSL1 PE=1 SV=1
Cae003852	ABCC9_HUMAN ATP-binding cassette sub-family C member 9 OS=Homo sapiens GN=ABCC9 PE=1 SV=2
Cae004455	CAC1C_RABIT Voltage-dependent L-type calcium channel subunit alpha-1C OS=Oryctolagus cuniculus GN=CACNA1C PE=1 SV=1
Cae007114	PF21B_MOUSE PHD finger protein 21B OS=Mus musculus GN=Phf21b PE=2 SV=1
Cae007700	SMCA4_HUMAN Transcription activator BRG1 OS=Homo sapiens GN=SMARCA4 PE=1 SV=2
Cae007800	RGPA2_HUMAN Ral GTPase-activating protein subunit alpha-2 OS=Homo sapiens GN=RALGAPA2 PE=1 SV=2
Cae008880	SYNG1_BOVIN Synapse differentiation-inducing gene protein 1 OS=Bos taurus GN=SYNDIG1 PE=2 SV=1
Cae010300	TRM44_HUMAN Probable tRNA (uracil-O(2)-)-methyltransferase OS=Homo sapiens GN=METTL19 PE=2 SV=2
Cae010642	XYLT1_HUMAN Xylosyltransferase 1 OS=Homo sapiens GN=XYLT1 PE=1 SV=1
Cae012317	IGF1R_HUMAN Insulin-like growth factor 1 receptor OS=Homo sapiens GN=IGF1R PE=1 SV=1
Cae015809	LTBP4_HUMAN Latent-transforming growth factor beta-binding protein 4 OS=Homo sapiens GN=LTBP4 PE=1 SV=2
Cae016408	UBX11_HUMAN UBX domain-containing protein 11 OS=Homo sapiens GN=UBXN11 PE=2 SV=2
Cae016846	ASHWN_BOVIN Ashwin OS=Bos taurus PE=2 SV=1
Cae017561	COBA1_MOUSE Collagen alpha-1(XI) chain OS=Mus musculus GN=Col11a1 PE=1 SV=2
Cae019371	DEDD2_MOUSE DNA-binding death effector domain-containing protein 2 OS=Mus musculus GN=Dedd2 PE=2 SV=1
Cae019889	PTBP1_PIG Polypyrimidine tract-binding protein 1 OS=Sus scrofa GN=PTBP1 PE=2 SV=1
Cae021496	WDR86_HUMAN WD repeat-containing protein 86 OS=Homo sapiens GN=WDR86 PE=2 SV=3

Sequence alignments of both wild and domestic PSGs are available in Additional file2

Supplementary Table S13. Positive selected genes (PSGs) in domestic goat

Gene	Annotation
GOAT_ENSBTAP00000004752	HPBP1_RAT Hsp70-binding protein 1 OS=Rattus norvegicus GN=Hspbp1 PE=2 SV=1
GOAT_ENSBTAP00000006565	CCKAR_HUMAN Cholecystokinin receptor type A OS=Homo sapiens GN=CCKAR PE=1 SV=1
GOAT_ENSBTAP00000010688	CHD5_HUMAN Chromodomain-helicase-DNA-binding protein 5 OS=Homo sapiens GN=CHD5 PE=1 SV=1
GOAT_ENSBTAP00000012776	FBX15_BOVIN F-box only protein 15 OS=Bos taurus GN=FBXO15 PE=2 SV=1
GOAT_ENSBTAP00000015790	WASH1_HUMAN WAS protein family homolog 1 OS=Homo sapiens GN=WASH1 PE=1 SV=1
GOAT_ENSBTAP00000016314	TB22A_MACFA TBC1 domain family member 22A (Fragment) OS=Macaca fascicularis GN=TBC1D22A PE=2 SV=2
GOAT_ENSBTAP00000017168	MMP15_MOUSE Matrix metalloproteinase-15 OS=Mus musculus GN=Mmp15 PE=2 SV=1
GOAT_ENSBTAP00000017556	LRP1_HUMAN Prolow-density lipoprotein receptor-related protein 1 OS=Homo sapiens GN=LRP1 PE=1 SV=1
GOAT_ENSBTAP00000020833	K1881_HUMAN Protein KIAA1881 OS=Homo sapiens GN=KIAA1881 PE=2 SV=2
GOAT_ENSBTAP00000021901	
GOAT_ENSBTAP00000024282-D2	CP2F3_CAPHI Cytochrome P450 2F3 OS=Capra hircus GN=CYP2F3 PE=2 SV=1
GOAT_ENSBTAP00000024433	T132A_HUMAN Transmembrane protein 132A OS=Homo sapiens GN=TMEM132A PE=2 SV=1
GOAT_ENSBTAP00000024536	NAV2_HUMAN Neuron navigator 2 OS=Homo sapiens GN=NAV2 PE=1 SV=2
GOAT_ENSBTAP00000024830	TMC6_HUMAN Transmembrane channel-like protein 6 OS=Homo sapiens GN=TMC6 PE=1 SV=2
GOAT_ENSBTAP00000024865	BRAC_HUMAN Brachyury protein OS=Homo sapiens GN=T PE=2 SV=1
GOAT_ENSBTAP00000026300	CD276_HUMAN CD276 antigen OS=Homo sapiens GN=CD276 PE=1 SV=1
GOAT_ENSBTAP00000026348	PALM_PIG Paralemmin OS=Sus scrofa GN=PALM PE=2 SV=1
GOAT_ENSBTAP00000031823	RCN3_BOVIN Reticulocalbin-3 OS=Bos taurus GN=RCN3 PE=2 SV=1
GOAT_ENSBTAP00000042804	ACHE_BOVIN Acetylcholine receptor subunit epsilon OS=Bos taurus GN=CHRNE PE=2 SV=1
GOAT_ENSBTAP00000045577	ADAD2_HUMAN Adenosine deaminase domain-containing protein 2 OS=Homo sapiens GN=ADAD2 PE=2 SV=1
GOAT_ENSBTAP00000052914	FAS_BOVIN Fatty acid synthase OS=Bos taurus GN=FASN PE=2 SV=1
GOAT_ENSBTAP00000053419	MGT5B_HUMAN Alpha-1,6-mannosylglycoprotein 6-beta-N-acetylglucosaminyltransferase B OS=Homo sapiens GN=MGAT5B PE=2 SV=2
GOAT_ENSBTAP00000053550	RBM33_HUMAN RNA-binding protein 33 OS=Homo sapiens GN=RBM33 PE=1 SV=3
GOAT_ENSBTAP00000053560	NFRKB_HUMAN Nuclear factor related to kappa-B-binding protein OS=Homo sapiens GN=NFRKB PE=1 SV=2
GOAT_ENSBTAP00000053740	RRBP1_HUMAN Ribosome-binding protein 1 OS=Homo sapiens GN=RRBP1 PE=1 SV=4

GOAT_ENSP00000164024	CELR3_HUMAN Cadherin EGF LAG seven-pass G-type receptor 3 OS=Homo sapiens GN=CELSR3 PE=1 SV=2
GOAT_ENSP00000243776	CHSS2_HUMAN Chondroitin sulfate synthase 2 OS=Homo sapiens GN=CHPF PE=1 SV=1
GOAT_ENSP00000245934	SYMPK_HUMAN Symplekin OS=Homo sapiens GN=SYMPK PE=1 SV=2
GOAT_ENSP00000259351	RGPS1_HUMAN Ras-specific guanine nucleotide-releasing factor RalGPS1 OS=Homo sapiens GN=RALGPS1 PE=1 SV=1
GOAT_ENSP00000263377	BRD4_HUMAN Bromodomain-containing protein 4 OS=Homo sapiens GN=BRD4 PE=1 SV=2
GOAT_ENSP00000283025	TEKT5_BOVIN Tektin-5 OS=Bos taurus GN=TEKT5 PE=2 SV=1
GOAT_ENSP00000290597	AL4A1_BOVIN Delta-1-pyrroline-5-carboxylate dehydrogenase, mitochondrial OS=Bos taurus GN=ALDH4A1 PE=2 SV=1
GOAT_ENSP00000300954	PCSK4_HUMAN Proprotein convertase subtilisin/kexin type 4 OS=Homo sapiens GN=PCSK4 PE=2 SV=1
GOAT_ENSP00000324560	ULK1_HUMAN Serine/threonine-protein kinase ULK1 OS=Homo sapiens GN=ULK1 PE=1 SV=1
GOAT_ENSP00000329846	GALT9_HUMAN Polypeptide N-acetylgalactosaminyltransferase 9 OS=Homo sapiens GN=GALNT9 PE=2 SV=3
GOAT_ENSP00000332287	SNG1_RAT Synaptogyrin-1 OS=Rattus norvegicus GN=Syngr1 PE=2 SV=1
GOAT_ENSP00000345931	CALU_HUMAN Calumenin OS=Homo sapiens GN=CALU PE=1 SV=2
GOAT_ENSP00000352995	ARHGI_HUMAN Rho guanine nucleotide exchange factor 18 OS=Homo sapiens GN=ARHGEF18 PE=1 SV=2
GOAT_ENSP00000354952	GAB2_HUMAN GRB2-associated-binding protein 2 OS=Homo sapiens GN=GAB2 PE=1 SV=1
GOAT_ENSP00000374121	INT1_MOUSE Integrator complex subunit 1 OS=Mus musculus GN=Ints1 PE=1 SV=2
GOAT_ENSP00000377721	COT2_BOVIN COUP transcription factor 2 OS=Bos taurus GN=NR2F2 PE=2 SV=1
GOAT_ENSP00000385631	SGSM1_MOUSE Small G protein signaling modulator 1 OS=Mus musculus GN=Sgsm1 PE=2 SV=2
GOAT_ENSP00000393795	Z512B_HUMAN Zinc finger protein 512B OS=Homo sapiens GN=ZNF512B PE=1 SV=1
GOAT_ENSP00000394978	LHX2_MOUSE LIM/homeobox protein Lhx2 OS=Mus musculus GN=Lhx2 PE=2 SV=1
GOAT_ENSP00000402044	ASB2_BOVIN Ankyrin repeat and SOCS box protein 2 OS=Bos taurus GN=ASB2 PE=2 SV=1
GOAT_ENSP00000403684	SMTN_HUMAN Smoothelin OS=Homo sapiens GN=SMTN PE=1 SV=5
GOAT_ENSP00000409544	CLD4_BOVIN Claudin-4 OS=Bos taurus GN=CLDN4 PE=2 SV=1
GOAT_ENSP00000410257	SCN5A_MOUSE Sodium channel protein type 5 subunit alpha OS=Mus musculus GN=Scn5a PE=2 SV=1
GOAT_ENSP00000424776	5HT3A_HUMAN 5-hydroxytryptamine receptor 3A OS=Homo sapiens GN=HTR3A PE=1 SV=1
goat_GLEAN_10011857	LHPP_BOVIN Phospholysine phosphohistidine inorganic pyrophosphate phosphatase OS=Bos taurus GN=LHPP PE=1 SV=1
goat_GLEAN_10014676	SNIP_RAT p130Cas-associated protein OS=Rattus norvegicus GN=P140 PE=1 SV=1
goat_GLEAN_10015977	CLIP2_MOUSE CAP-Gly domain-containing linker protein 2 OS=Mus musculus GN=Clip2 PE=1 SV=2

Supplementary Table S14. Positive selected genes associated with nervous system

Gene	Annotation
**wild goat**	
Cae003852	ABCC9_HUMAN ATP-binding cassette sub-family C member 9 OS=Homo sapiens GN=ABCC9 PE=1 SV=2
Cae004455	CAC1C_RABIT Voltage-dependent L-type calcium channel subunit alpha-1C OS=Oryctolagus cuniculus GN=CACNA1C PE=1 SV=1
Cae008880	SYNG1_BOVIN Synapse differentiation-inducing gene protein 1 OS=Bos taurus GN=SYNDIG1 PE=2 SV=1
Cae016846	ASHWN_BOVIN Ashwin OS=Bos taurus PE=2 SV=1
**domestic goat**	
GOAT_ENSBTAP00000006565	CCKAR_HUMAN Cholecystokinin receptor type A OS=Homo sapiens GN=CCKAR PE=1 SV=1
GOAT_ENSBTAP00000010688	CHD5_HUMAN Chromodomain-helicase-DNA-binding protein 5 OS=Homo sapiens GN=CHD5 PE=1 SV=1
GOAT_ENSBTAP00000024433	T132A_HUMAN Transmembrane protein 132A OS=Homo sapiens GN=TMEM132A PE=2 SV=1
GOAT_ENSBTAP00000024536	NAV2_HUMAN Neuron navigator 2 OS=Homo sapiens GN=NAV2 PE=1 SV=2
GOAT_ENSBTAP00000026348	PALM_PIG Paralemmin OS=Sus scrofa GN=PALM PE=2 SV=1
GOAT_ENSBTAP00000042804	ACHE_BOVIN Acetylcholine receptor subunit epsilon OS=Bos taurus GN=CHRNE PE=2 SV=1
GOAT_ENSBTAP00000045577	ADAD2_HUMAN Adenosine deaminase domain-containing protein 2 OS=Homo sapiens GN=ADAD2 PE=2 SV=1
GOAT_ENSBTAP00000053419	MGT5B_HUMAN Alpha-1,6-mannosylglycoprotein 6-beta-N-acetylglucosaminyltransferase B OS=Homo sapiens GN=MGAT5B PE=2 SV=2
GOAT_ENSP00000283025	TEKT5_BOVIN Tektin-5 OS=Bos taurus GN=TEKT5 PE=2 SV=1
GOAT_ENSP00000324560	ULK1_HUMAN Serine/threonine-protein kinase ULK1 OS=Homo sapiens GN=ULK1 PE=1 SV=1
GOAT_ENSP00000329846	GALT9_HUMAN Polypeptide N-acetylgalactosaminyltransferase 9 OS=Homo sapiens GN=GALNT9 PE=2 SV=3
GOAT_ENSP00000332287	SNG1_RAT Synaptogyrin-1 OS=Rattus norvegicus GN=Syngr1 PE=2 SV=1
GOAT_ENSP00000393795	Z512B_HUMAN Zinc finger protein 512B OS=Homo sapiens GN=ZNF512B PE=1 SV=1
GOAT_ENSP00000394978	LHX2_MOUSE LIM/homeobox protein Lhx2 OS=Mus musculus GN=Lhx2 PE=2 SV=1
GOAT_ENSP00000424776	5HT3A_HUMAN 5-hydroxytryptamine receptor 3A OS=Homo sapiens GN=HTR3A PE=1 SV=1
goat_GLEAN_10015977	CLIP2_MOUSE CAP-Gly domain-containing linker protein 2 OS=Mus musculus GN=Clip2 PE=1 SV=2

Supplementary Table S15. Statistics of pseudo-chromosome assembly

Chromosome	Domestic goat(chr1-X chromosome); bovine (Y chromosome) (bp)	Wild goat (bp)
chr1	155,011,307	157,391,697
chr10	99,198,151	107,481,852
chr11	105,305,221	102,029,473
chr12	83,600,723	84,959,673
chr13	80,625,018	83,174,693
chr14	92,307,899	93,701,077
chr15	78,986,926	79,226,036
chr16	77,678,508	80,957,510
chr17	71,877,758	72,094,808
chr18	61,069,492	67,556,651
chr19	62,130,014	68,039,774
chr2	135,415,751	128,021,631
chr20	71,280,158	69,975,700
chr21	66,773,250	71,174,368
chr22	57,956,300	61,116,191
chr23	49,403,180	51,750,272
chr24	61,756,751	62,254,493
chr25	41,496,684	49,030,443
chr26	50,169,583	53,791,435
chr27	44,118,849	46,295,624
chr28	43,231,948	38,538,704
chr29	48,376,377	49,577,580
chr3	116,796,116	114,356,265
chr4	115,961,478	118,084,723
chr5	111,056,284	114,006,803
chr6	114,334,461	124,181,624
chr7	106,547,263	111,971,386
chr8	111,020,524	117,294,191
chr9	90,294,081	91,316,209
chrX	121,952,852	123,475,178
chrY	43,300,181	17,295,773

Supplementary Table S16. Genes in the wild goat Y psedu-chromosome

Gene ID	Start site	End site	Gene Annotation
Cae019504*	1624632	1626537	KALM_HUMAN Anosmin-1
Cae014288	3116517	3116888	troponin I type 1 (skeletal, slow); K10371 troponin I, slow skeletal muscle
Cae014289	3164299	3231449	ASMT_BOVIN Acetylserotonin O-methyltransferase
Cae014290	3220065	3538548	DHRSX_HUMAN Dehydrogenase/reductase SDR family member on chromosome X
Cae014291	3615522	3615863	troponin I type 1 (skeletal, slow); K10371 troponin I, slow skeletal muscle
Cae014292	3644998	3651109	DHRSX_HUMAN Dehydrogenase/reductase SDR family member on chromosome X
Cae014293	3677163	3677861	ZBED1_HUMAN Zinc finger BED domain-containing protein 1
Cae014294	3798744	3821926	CD99_HUMAN CD99 antigen
Cae014295	3908182	3926485	ARSD_HUMAN Arylsulfatase D
Cae014296	3981441	3994667	ARSH_CANFA Arylsulfatase H
Cae014297	4084180	4084383	
Cae014298	4087818	4115576	MXRA5_HUMAN Matrix-remodeling-associated protein 5
Cae023005	6154453	6156702	SHRM2_HUMAN Protein Shroom2
Cae022233	6207113	6207772	MAGH1_HUMAN Melanoma-associated antigen H1
Cae022868	6787028	6787270	NU1M_SHEEP NADH-ubiquinone oxidoreductase chain 1
Cae022869	6794907	6795203	NU4LM_CAPHI NADH-ubiquinone oxidoreductase chain 4L
Cae022205*	6843987	6848335	PRAME_HUMAN Melanoma antigen preferentially expressed in tumors
Cae022204	6889873	6892675	
Cae021801	6923529	6931543	TRI64_HUMAN Tripartite motif-containing protein 64
Cae021802	6943275	6948760	TRI43_HUMAN Tripartite motif-containing protein 43
Cae021803	6956633	6957805	UBFL1_HUMAN Putative upstream-binding factor 1-like protein 1
Cae021804	6960169	6962178	
Cae021805*	6973986	6976728	PRA24_HUMAN Putative PRAME family member 24
Cae021806	7008132	7008572	TRI43_HUMAN Tripartite motif-containing protein 43
Cae021807	7009591	7011001	TR64C_HUMAN Putative tripartite motif-containing protein 64C
Cae021808	7036341	7041505	TRI64_HUMAN Tripartite motif-containing protein 64
Cae020167	7189678	7189905	SHRM2_RAT Protein Shroom2

Cae020166*	7388673	7395490	PRAME_HUMAN Melanoma antigen preferentially expressed in tumors
Cae022671*	7589679	7598536	TSPY1_BOVIN Testis-specific Y-encoded protein 1
Cae022600*	7695782	7696315	PRA12_HUMAN PRAME family member 12
Cae021280	7742604	7764583	PRDM9_HUMAN Histone-lysine N-methyltransferase PRDM9
Cae021279	7899106	7899635	
Cae021767*	7991581	7993132	Z280B_HUMAN Zinc finger protein 280B
Cae021768*	8003417	8010771	TSPY1_BOVIN Testis-specific Y-encoded protein 1
Cae021748	8317762	8341328	LIPA1_HUMAN Liprin-alpha-1
Cae023032*	8377980	8378951	Z280B_HUMAN Zinc finger protein 280B
Cae014147	8632438	8633550	YBOX1_RABIT Nuclease-sensitive element-binding protein 1
Cae014148	8709716	8710492	SPIN2_PONAB Spindlin-2
Cae014149	8768864	8769332	DCC1_HUMAN Sister chromatid cohesion protein DCC1
Cae014150	8824016	8824792	SPIN2_BOVIN Spindlin-2
Cae014151	8976223	8976396	MED31_XENTR Mediator of RNA polymerase II transcription subunit 31
Cae014152	9175330	9175611	TX264_HUMAN Testis-expressed sequence 264 protein
Cae014153	9259213	9261072	UBQL2_HUMAN Ubiquilin-2
Cae014154	9480747	9485261	KLF8_HUMAN Krueppel-like factor 8
Cae014155	9761483	9800263	RRAGB_HUMAN Ras-related GTP-binding protein B
Cae018359	10091731	10104688	
Cae018360	10353263	10353436	COX1_TETNG Cytochrome c oxidase subunit 1
Cae018361	10475480	10482579	ANR26_HUMAN Ankyrin repeat domain-containing protein 26
Cae018362	10597052	10597734	OR5W2_HUMAN Olfactory receptor 5W2
Cae018878	11404311	11404706	
Cae022763	11593943	11594311	G3N2L4_BOVIN Uncharacterized protein
Cae019830*	12153317	12153664	Z280A_HUMAN Zinc finger protein 280A
Cae020602*	12677666	12678355	SRY_CAPHI Sex-determining region Y protein
Cae006770	14525302	14614781	CTNA3_HUMAN Catenin alpha-3
Cae006769	14919740	14925414	CTNA3_HUMAN Catenin alpha-3
Cae006768	16243868	16247173	RPP30_BOVIN Ribonuclease P protein subunit p30
Cae021003	16870130	16876142	FAM3A_PONAB Protein FAM3A
Cae021002	16888864	16899927	G6PD_CRIGR Glucose-6-phosphate 1-dehydrogenase
Cae021001	16907020	16919651	NEMO_BOVIN NF-kappa-B essential modulator

Cae021000	17128853	17129215	PRDM9_HUMAN Histone-lysine N-methyltransferase PRDM9

*** Homologs of male specific region of Y chromosome (MSY) gene[2].**

Supplementary Table S17. Information about five re-sequencing Australian goat sample.

(a) Characteristics of goat breeds sampled

Sample_Name	AUST_RL_100	AUST_RL_200	AUST_Boer_942	AUST_Boer_P439	AUST_Cash_E040
Breed	Rangeland	Rangeland	Boer	Boer	Cashmere
Sex	Intersex	Intersex	Female	Female	Female
Poll/Horn	Polled	Polled	Horned	Polled	Polled
Country	Australia	Australia	Australia	Australia	Australia
Longitude	*144°15'E*	*144°15'E*	*151°50'E*	*151°50'E*	*152°34'E*
Latitude	*32°53'S*	*32°53'S*	*27°17'S*	*27°17'S*	*27°31'S*
Sampling Date	early December 2011	early December 2011	late November 2011	late November 2011	late November 2011
Closest Locality	Ivanhoe	Ivanhoe	Goombungee	Goombungee	Tarampa
Closest City	Dubbo	Dubbo	Toowoomba	Toowoomba	Ipswich

(b) Statistics of re-sequencing results

Sample ID	No.of Single Reads	No. of base pairs	Coverage(X)
AUST_RL_100	180,877,300	18,087,730,000	6.237148276
AUST_RL_200	185,635,904	18,563,590,400	6.401238069
AUST_Boer_942	233,734,198	23,373,419,800	8.059799931
AUST_Boer_P439	231,491,956	23,149,195,600	6.654021172
AUST_Cash_E040	192,966,614	19,296,661,400	7.982481241

Supplementary Table S18. Candidate deleted gene copies in domestic goats

mapped gene	Gene symbol	s100-Rangeland goat	s200-Rangeland goat	942a-Australian Boer goat	P439-Australian Boer goat	E040-Australian Cashmere goat	Yunling black goat	Bamu wild goat	Khonj wild goat	Blastp identity(%)
Cae022887	MYADM	0.008[a]	0.017	0.006	0.002	0.02	0.016	0.81	1.20	88.81
Cae022711	MYADM	0.038	0.136	0.048	0.12	0.013	0.018	0.99	0.51	80.78
Cae022915	MYADM	0.086	0.023	0.159	0.086	0.093	0.07	1.168	0.93	83.97
Cae021087	ABCC4	0.066	0.004	0.11	0.001	0.006	0.006	0.958	0.41	83.78
Cae019751	ABCC4	0.115	0.015	0.083	0.126	0.145	0.026	1.122	0.83	94.56
Cae019750	ABCC4	0.102	0.009	0.076	0.006	0.074	0.059	1.37	0.79	91.89
Cae021766	BTN1A1	0	0.095	0.099	0	0.13	0.001	0.818	1.21	94.23
Cae020166	PRAME	0.165	0	0	0	0	0	0.968	1.06	42.34
Cae022357	KIR3DL1	0.171	0.164	0.029	0	0.089	0.171	0.982	1.02	66.1
Cae022610	WC1.1	0.09	0.14	0.133	0.09	0.113	0.169	0.876	0.93	78.52

a. Value in Column 3-10 represents CNV ratio: read depth (RD)/one average fold depth in reference

Supplementary Table S19. Candidate gained gene copies in domestic goats

mapped gene	Gene Symbol	s100-Rangeland goat[a]	s200-Rangeland goat	942a-Australian Boer goat	P439-Australian Boer goat	E040-Australian Cashmere goat	Yunling black goat	Bamu wild goat	Khonj wild goat
Cae022930	LGALS9B	2.789	2.247	3.331	2.173	2.68	3.539	1.05	1.05
Cae022695	CFH	3.839	3.423	2.101	1.91	2.852	4.449	1.146	1.17
Cae022538	LOC777601	2.03	5.617	4.599	3.532	4.096	4.258	1.066	0.99
Cae020183	DOCK11	2.427	3.25	3.929	4.372	4.147	4.773	1.02	1.05
Cae016923	HELLS	2.084	2.435	2.103	2.471	2.066	2.571	1.172	0.68
Cae013714	NBEA	2.199	1.983	2.211	1.933	2.08	2.32	0.894	0.31
Cae012687	TRIM5	3.036	1.816	1.911	2.055	1.927	3.351	1.019	0.63
Cae011519	CBX3	4.224	2.157	2.4	4.986	2.69	3.891	0.976	0.81
Cae010935	RFX3	2.164	2.046	2.311	2.389	2.649	2.241	1.023	0.91
Cae009652	PI4K2B	2.35	1.859	1.901	1.863	1.976	2.495	0.855	0.87
Cae009178	PLXDC2	2.378	2.499	1.863	2.384	2.114	2.55	1.179	1.43
Cae003870	NR3C2	3.23	2.474	1.893	3.164	2.199	2.081	1.093	1.28
Cae003743	ERC2	2.931	1.934	2.576	1.889	2.325	3.017	1.19	0.78
Cae001260	G3BP1	3.098	3.094	3.239	3.226	2.232	3.412	1.174	1.27
Cae001070	PMAIP1	2.728	2.358	2.242	3.434	2.52	3.618	1.019	0.47
Cae000639	MAGI1	2.158	2.446	2.103	2.712	2.483	3.262	0.956	0.45
Cae000243	GABRB2	2.226	2.071	2.293	2.54	2.062	2.75	0.918	1.01
Cae000155	Npas3	1.915	2.383	1.985	2.49	2.268	2.898	0.985	1.43

a. Value in Column 3-10 represents CNV ratio: read depth (RD)/one average fold depth in reference

Supplementary Table S20. Classification of representative candidate copy gain and loss genes in domestic goat based on functions.

Function	Gene
**Production-trait related**	
candidate loss in domestic goat	*MYADM, BTN1A1, PRAME*
**Immune Response**	
candidate loss in domestic goat	*ABCC4,PRAME,WC1.1,KIR3DL1*
candidate gain in domestic goat	*CFH,TRIM5*
**Nervous-system related**	
candidate gain in domestic goat	*ERC2,GABRB2,Npas3*

Supplementary Table S21. Copy number variations of color genes among wild goat and domestic goat breeds

Gene	s100-Rangeland goat[a]	s200-Rangeland goat	942a-Australian Boer goat	P439-Australian Boer goat	E040-Australian Cashmere goat	Yunling Black goat	Bamu wild goat	Khonj wild goat
ASIP	1.328	1.008	2.075	2.383	1.984	0.85	1.004	0.878
SLC7A11	3.194	4.293	4.262	4.013	4.537	3.84	5.888	3.582
OCA2	1.628	1.871	2.171	1.799	2.006	1.569	2.493	0.494
MITF	2.615	2.714	3.529	2.708	3.178	2.62	3.429	1.58
ATRN	1.837	2.438	1.567	1.736	3.027	2.581	1.648	0.300
FIG4	5.657	4.97	5.615	5.375	5.136	4.122	5.044	2.26
GNAQ	4.364	3.837	4.181	3.479	3.474	3.813	5.56	1.701
HELLS	2.084	2.435	2.103	2.066	2.471	2.571	1.172	0.680
MUTED	0	0	0.501	0.42	0.29	0.635	0.935	0.950
OSTM1	2.89	3.093	3.239	2.922	2.732	2.786	2.317	2.23
TRPM7	4.637	5.13	4.525	4.479	4.802	4.254	6.24	2.035
VPS33A	3.073	2.437	2.454	2.596	2.934	2.47	3.129	1.445
Adamts20	3.848	3.951	3.951	4.019	3.579	4.334	3.857	1.773

a. Value in Column 2-9 represents CNV ratio: read depth (RD)/one average fold depth in reference

Supplementary Table S22. qPCR primers used for the verification of five picked candidate gained gene copies in domestic goats.

Gene name	Primer	Sequence
TRIM5	F	GGAGATTGCACCACAGTACC
TRIM5	R	CTCTCGGCCCCATATGTCC
ASIP	F	CAATGCTAGGCTCTAATACCC
ASIP	R	GAATTTGGTTTAACTTCGTGT
Npas3	F	GAAAACTATATGCGGCCCTC
Npas3	R	GAGTACAAGCCCCATTGCTA
CFH	F	GTTTCGTATTTTATGGCTTG
CFH	R	CTGGCTATTTATTCATAGGAAC
GABRB2	F	CTGCCCTAAATAGTCCAGC
GABRB2	R	ATTACTTTACTAGCGTGTGAG
C7orf28b	F	GATCTACTCGACATATTTGGTG
C7orf28b	R	GCAGTGTACTTGACTATACTCA

Supplementary Table S23. The comparison of gene expression level between one wild goat brain tissue and one Cashmere goat brain tissue, for the candidate gain and loss genes in domestic goats.

Gene name	Gene symbol	Gene copies in domestic goats	Brain FPKM in wild goat	Brain FPKM in cashmere goat	Folds change
Cae022930	LGALS9B	gained	0.0	0.5	>>2
Cae022695	CFH	gained	0.0	0.4	>>2
Cae022538	LOC777601	gained	0.0	0.0	NA
Cae020183	DOCK11	gained	1.2	2.5	2.1
Cae016923	HELLS	gained	0.8	1.1	1.4
Cae013714	NBEA	gained	22.9	64.8	2.8
Cae012687	TRIM5	gained	0.0	1.1	>>2
Cae011519	CBX3	gained	173.3	221.3	1.3
Cae010935	RFX3	gained	4.9	4.8	1.0
Cae009652	PI4K2B	gained	0.9	2.2	2.5
Cae009178	PLXDC2	gained	4.3	9.0	2.1
Cae003870	NR3C2	gained	5.6	22.0	3.9
Cae003743	ERC2	gained	83.2	54.6	0.7
Cae001260	G3BP1	gained	47.4	48.3	1.0
Cae001070	PMAIP1	gained	0.0	0.0	NA
Cae000639	MAGI1	gained	11.2	22.3	2.0

Cae000243	GABRB2	gained	7.3	101.2	13.9
Cae000155	NPAS3	gained	5.3	14.1	2.7
Cae008233	ASIP	gained in Cashmere goats	76.9	31038.7	403.4
Cae022887	MYADM	deleted	0.0	0.0	NA
Cae022711	MYADM	deleted	1.4	0.0	<<-2
Cae022915	MYADM	deleted	0.0	0.0	NA
Cae021087	ABCC4	deleted	0.0	0.5	>>2
Cae019751	ABCC4	deleted	0.0	0.0	NA
Cae019750	ABCC4	deleted	0.0	0.0	NA
Cae021766	BTN1A1	deleted	0.0	0.0	NA
Cae020166	PRAME	deleted	0.0	0.0	NA
Cae022357	KIR3DL1	deleted	0.0	0.0	NA
Cae022610	WC1.1	deleted	0.0	0.0	NA

3 Supplementary Methods

3.1 Data Generation

3.1.1 Sequencing

DNA sample from a male bezoar (*Capra aegagrus*) from Bamu National Park was collected. Bamu National Park is located at the northwest of the city of Shiraz in Fars. Bamu wild goat sample was used to construct short insert sequencing libraries and mate-pair libraries (including insert size of 250 bp, 500 bp, 800 bp, 2kb, and 5kb). Due to the scarcity of this wild goat sample, in order to improve scaffolding in genome assembly, we further used three more wild goats' DNA to construct mate pair sequencing libraries (10kb and 20kb). The three bezoars were from Khonj, Sabzevar, Farashband, Iran, respectively. Based on this strategy, the Bamu Park wild goat was the only one used to construct contigs in the final assembly while other individuals only contributed to scaffolding. Collection of these samples obtained official certificate of legal acquisition of the wild goat sample from the Iranian authority, and the certificate is available upon request.

Following Illumina standard sequencing protocol, DNA of specific length was gel purified after electrophoresis and ligated with adapter. All the libraries were sequenced with Illumina Hiseq 2000 sequencers at BGI-Shenzhen. In total, 381.50 Gb sequencing data with 136.26 sequence coverage was generated (Supplementary Table S1). The usage for each wild goat sample was also listed in Supplementary Table S1.

DNA sequencing from two Australian feral Rangeland goats, two Boer goats and one Cashmere goat were performed on illumina second-generation sequencing platform. Background and sequencing information of each resequencing sample was shown in Supplementary Table S17.

3.1.2 Filtering raw data

Before analysis of the sequencing data, it is essential to take a series of checking and filtering

raw reads since sequencing errors or contamination cannot be completely avoided and may cause bias in later assembly. The filtering criteria following are applied[3].

(1) Removed reads which contained ambiguous character N or poly A more than 10% of short insert-size reads or more than 20% of long insert-size ones.

(2) Removed low quality reads which have more than 40 bases with quality score less than 5 for short insert-size libraries and 30 bases for the long ones. Quality score here refers to the error probability of base-calling, e.g Q20 is defined as an error probability of 0.01.

(3) Trimmed off the head and tail of all short insert-size reads. (cut off length varied based on quality score distribution. (Supplementary Table S1)

(4) Removed reads with adapter contamination, which more than 10bp aligned to the adapter sequence (allowing less than or equal to 3bp mismatch).

(5) Removed short insert-size reads in which Read1 and Read2 overlapped more than or equal to 10bp allowing 10% mismatch. Read1 and Read2 refer to forward and reverse ends of one PE reads.

(6) Filter PCR duplicate .When Read1 and Read2 of two paired end reads are totally identical, these reads can be considered duplicate.

Applying such criteria, 38.7% of raw data were filtered out. Thus we got 233.90-Gb clean data with 83.54-fold sequence coverage (Supplementary Table S1).

3.1.3 Kmer Analysis

Before sequence assembly, clean reads were used for Kmer analysis to get a glance of the characteristic of assembly from the sequencing reads[3]. The kmer here refers to a substring of length k divided artificially from sequencing reads. The frequency of each kmer can be calculated from the sequencing reads. Theoretically, the kmer frequencies along its depth gradient follow a Poisson distribution. By deduction, we can calculate the genome size from this formulation: G=K_num/Peak_depth, where the K_num is the total number of kmer, and

Peak_depth is the expected value of kmer depth. Typically, K=17.

Total 773,730,862 reads with sequencing coverage of 34X from the short insert size (500bp and 800bp) libraries were selected for 17mer-analysis (Supplementary Table S2). Our program incorporated the fast, parallel k-mer counter – Jellyfish[4] using parameter: -t 50 -k 17. The 17-mer frequency distribution derived from the sequencing reads was plotted in Supplementary Fig. S2, the peak of the 17-mer distribution is about 30, and the total number of K-mer is 87,100,255,318, then the genome size can be estimated by the formula (G=K_num/Peak_depth) as 2.90Gb.

The k-mer distribution (Supplementary Fig. S2) can also be applied for roughly estimating heterozygous rate and repeat rate. If the heterozygous rate is higher, then a small peak will be presented at 1/2 of Peak_depth. If this genome contains high proportion of repeat, the distribution will display a fat tail which indicates more than expect proportion of the genome have a high sequencing depth which may due to sequence similarly.

In general, the genome size is estimated about 2.90 Gb. There is no significant heterozygosis but there're some repeat content in this genome.

3.1.4 Genome assembly

The wild goat genome was assembled by SOAPdenovo[5] software, which is based on the *de Bruijn* graph algorithm. The assembly mainly follows these steps:

Firstly, lower frequency kmer were excluded and the remaining reads were used to construct *de Bruijn* graph. Then according to the set parameters “pregraph -K 49 -d 3 -R”, the graph was simplified by removing tips, merging bubbles, deleting the low coverage of the connection and the small repeat. Finally, the contig sequences were genertated by connecting the k-mer path with contig N50 1,602 bp, N90 194 bp, and total length 2,450,210,404 bp (“contig -M 3”).

Secondly, the scaffolds were constructed by realigning all the usable reads onto the contig sequences by using parameters “map -k 37”. Based on the insert-size information of

paired-end reads with each ends aligned to different contigs, the distance between aligned contigs can be estimated. Thus aligned contigs could be connected by filling in gaps in this length using “scaff -F”. Integrating data from both short insert-size libraries and long insert-size ones, we finallly obtained scaffolds with N50 2,114,973bp, N90 427,992 bp, and total length 2,842,429,867 bp.

The next step is closing gap. To close the gaps inside the constructed scaffolds, we exploited the paired-end information to retrieve the read pairs that had one end mapped to the unique contigs and the other located in the gap region, then did a local assembly for these collected reads. In summary, 66.82% of the intra-scaffold gaps were closed, which stand for 51.51% of the sum gap length.

Finally, the draft genome assembly reached contig N50 size of 18,965 bp and scaffold N50 size of 2,057,686 bp. The total length of scaffolds region is 99% of the previously estimated genome size, which appreciably assured later genome annotation and further analysis.

3.1.5 Evaluation of the assembled genome sequence

We mainly used three methods to evaluate the quality of assembled genome.

(1) Mapping-based assessment

To verify the single-base accuracy of the assembled genome sequence, all the usable sequencing reads were realigned onto the assembled scaffolds using SOAPaligner [6]. The alignment result showed that the average coverage ratio of the scaffolds were 92%. Furthermore, the distribution of sequencing depth on the single-base level were calculated and found to fit the normal distribution roughly with peak depth at 69× as expected (Supplementary Fig. S3)

(2) GC content assessment

We also measured the GC content distribution to assess whether the sequencing was affected by GC-biased non-random sampling. The distribution is generated by using 500bp as non-overlapping sliding windows along the genome and then calculating the GC content of each window. With the plot (Supplementary Fig. S4), we can compare the GC content distribution

of the related species (*Homo sapiens, Bos taurus, Capra hircus*), which are supposed to have more similar profile. In the plot, the curve of wild goat shapes similarly with the one of domestic goat (*C.hir*), which significantly suggests the high quality of sequencing and assembly finished above.

(3) CEGMA assessment

CEGMA (Core Eukaryotic Genes Mapping Approach)[7] (http://korflab.ucdavis.edu/Datasets/cegma/, version 2.3, with parameter "--mam") was used to evaluate the gene space of the wild goat genome assembly, with the proportion of mapped CEGs (Core Eukaryotic Genes) corresponding to the gene space completeness of the assembly. All 248 CEGs chosen from six model species (*H. sapiens, D. melanogaster, A. thaliana, C. elegans, S. cerevisiae and S. pombe*) were characterized as house-keeping gene families and expected to be present in all eukaryotic genomes. The proteins of each CEG were mapped to the wild goat scaffold assembly (Supplementary Table S5). 97.98% core eukaryotic genes (http://korflab.ucdavis.edu/Datasets/cegma/) from six model organisms mapped to the assembled sequence of wild goat (Supplementary Table S5).

In conclusion, the assembled sequence can be considered as a high quality one as it is 99% complete and with an N50 scaffold length larger than gene size. Combined with the reasonable reads mapping quality, GC content distribution and appreciable CEGMA evaluation result, the draft assembly is a decent target for further genome annotation.

3.1.6 Anchoring scaffolds to chromosomes

Reference genome of domestic goat (CHIR_1.0) was obtained from NCBI and bovine Y chromosome was downloaded from NCBI (GenBank accession is NC_016145.1).

To anchor wild goat scaffolds onto chromosomes, we exploited their synteny relationship with domestic goat chromosomes. Firstly, we masked repeat sequences in both wild goat genome and domestic goat genome. We also filtered short wild goat scaffolds which are less than 2 Kb. Then, LASTZ[8] (version 1.01.50) was used to align scaffolds of wild goat to chromosomes of domestic goat with parameters "T=2 C=2 H=2000 Y=3400 L=6000 K=2200". We clustered the LASTZ hits within a window of 100 Kb and filtered orphan hit in

each window. A wild goat scaffold could be aligned to multiple domestic goat loci or chromosomes. We sorted the alignments by length. When the longest alignment is more than twice of the second in length, we consider it as the best alignment of this scaffold and link the wild goat's scaffold with the aligned loci in the domestic goat's chromosome. At last, we ordered and oriented all the linked wild goat scaffolds and constructed pseudo-chromosomes.

Wild goat Y chromosome was constructed following these pipeline. First, BLAT (version 34) [9] was used to align peptide sequence of bovine Y chromosome to unanchored wild goat scaffolds and all the wild goat scaffolds, respectively. We filtered the hits with a coverage rate of the protein length lower than 60%. Then, we mapped contigs of bovine Y chromosome to unanchored wild goat scaffolds and all the wild goat scaffolds respectively with LASTZ (parameters: T=2 C=2 H=2000 Y=3400 L=6000 K=2200). LASTZ hits would be clustered based on these standards: the distance between two hits is less than 1 Kb; or the distance is more than 1 Kb and less than 5 Kb with the unmapped region having more than 50% repeat sequences. In the same way, we sorted the alignments by length, and picked the longest alignment of each wild scaffold and linked them to bovine chromosome Y. Combined the result of BLAT and LASTZ, we constructed draft assembly of wild goat chromosome Y. In addition, we identified four scaffolds related to pseudoautosomal region and anchored them on the head of chromosome Y.

3.1.7 Repeat annotation

There are two main types of repeats in the genome. One is the tandem repeats with low complexity such as homopolymeric runs of nucleotides; the other is interspersed repeats, also named transposable elements.

We searched the genome for tandem repeats with the help of program Tandem Repeats Finder (TRF)[10], with parameters set to be “2 7 7 80 10 50 2000 -d -h”. Transposable elements (TEs) were identified in the genome by a combination of homology-based and de novo approaches. Homology-based approach involves commonly used databases of known repeats.

(1) Homolog based prediction: identification of known TEs

The known transposable elements (TEs) were identified based on the known repeat database

using corresponding software: RepeatMasker (http://repeatmasker.org), RepeatProteinMask. TEs in the genome assembly were identified at the DNA and protein level. RepeatMasker was applied for DNA-level identification using a custom library, with parameters “-norna, -lib”. At the protein level, RepeatProteinMask, updated software in the RepeatMasker package, was used to perform RMBlast against the TE protein database, with parameters “-pvalue 1e-4”.

(2)*De novo* repeat prediction

RepeatModeler (http://repeatmasker.org/) and LTR FINDER[11] were used in identifying *de novo* repeats inferred from the assembled genome. These programs predict repeats in different fashions:

(a) The RepeatModeler was used to construct a de novo wildgoat repeat library applying two *de novo* repeat-finding programs (RECON & RepeatScout). Using the default parameters, the generated results were consensus sequences and classification information of each repeat family. Then RepeatMasker was called again to run on the assembly genome, using the RepeatModeler consensus sequence as the library.

(b) LTR FINDER (parameters: “-w -s bosTau4-tRNAs.fa) searches the whole genome for a characteristic structure of the full-length long terminal repeat retrotransposons (LTRs), mainly based on that their ~18bp terminal sequences are complementary to the 3' tail of some tRNAs. Cattle tRNAs downloaded from http://lowelab.ucsc.edu/GtRNAdb were used as references.

3.1.8 Gene annotation

The process of finding genes aims to locate gene locus as well as its splicing sites. For gene prediction, we mainly used the homology-based method, the *de novo* method and Glean. Due to the close relationship between wild goat and domestic goat, we also applied ESTs and RNA-seq data of domestic goat to annotate gene.

(1) *Ab initio* prediction

De novo prediction was performed on the repeat-masked wild goat genome based on the HMM model. Programs applied including AUGUSTUS[12], GENSCAN[13] and GlimmerHMM[14, 15] with parameters trained for the wild goat genome. Short genes (CDS

length < 150bp) and low-quality genes (gaps covered more than 10% of the coding region) were discarded. Through this method, 20,195, 41,384 and 19,453 gene models were predicted, respectively.

(2) Homolog-based prediction

Homolog proteins of related species (*H. sapiens*, *B. taurus*, *C. hircus*, *B. grunniens* and *S. scrofa*) were mapped to the genome using BLAT (version 0.34, coverage > 0,5)[9]. The aligned sequence as well as its query protein were filtered by identity and coverage criteria and then passed to GeneWise (version 2.2.0, parameters "-sum -genesf -gff")[16] refine the spliced alignments.

(3) EST prediction

ESTs of domestic goat were aligned to the wild goat genome using BLAT (version 0.34, identity >0.95, coverage >0.90) to generate spliced alignments. The spliced alignments were clustered and assembled according to overlapping information using PASA[17] (http://pasa.sourceforge.net) with default parameters.

(4) RNA-Seq[18]

First, transcriptome reads of domestic goat were aligned against the wild goat genome using TopHat[19] (version 1.3.3 with bowtie v0.12.7 parameters: "--max-intron-length 300000, -m 2 –r insert-size --mate-std-dev STD --coverage-search --microexon-search") to identify candidate exon regions and the donor and acceptor sites. Then, Cufflinks[20] (version 1.1.0, parameters: "-I 300000") was performed to assemble the alignments into transcripts. Finally, based on these assembled candidate potential transcript sequences, ORF was predicted to get reliable transcripts by using HMM-based training parameter.

(5) Evidence integration

All genes predicted by methods mentioned above were clustered to create a final gene set. The clustering process consisted of the following steps:

(a) For homology-based gene models, those with short length, unusual N%, inner stops, less 80% coverage of the homology proteins, and different structural of the homology proteins were filtered. Then we merged these homology-based genes in the priority order of

human, cattle, goat, yak, pig. Overlapped genes were defined to be redundant and were filtered.

(b) For *de novo* gene models, those supported by both EST/unigene-based method and RNA transcripts analysis were retained. Then incorporated them in the homology-based gene set and filtered the redundant genes.

(c) Gathered all the evidences for GLEAN software[21] with default settings to predict gene structures. Its result was used to supplement the current gene set.

After a manual check, we annotated a total of 23,217 genes in the wild goat.

3.1.9 Gene function annotation

Gene functions were assigned according to the best match of the alignments using Blastp [22] to SwissProt and Translated EMBL Nucleotide Sequence Data Library (TrEMBL) databases[23]. The motifs and domains of genes were determined by InterProScan[24] against protein databases including ProDom, PRINTS, Pfam, SMART, PANTHER and PROSITE, and corresponding Gene Ontology (GO) [25] ID were obtained. All genes were aligned against KEGG[26] proteins using Blastp, and the pathway in which the gene might be involved was derived from the matched genes in KEGG.

3.1.10 ncRNA annotation

The tRNA genes were identified by tRNAscan-SE with appropriate default parameters. The C/D box snoRNAs were identified by Snoscan using appropriate parameters rRNA and appropriate parameters methylation sites. Other ncRNAs, including miRNA, snRNA, were identified using INFERNAL software by searching against the Rfam database with appropriate default parameters.

3.2 Analysis of gene families

3.2.1 Identification of wild goat gene families

To identify wild goat gene families, we selected the following reference species to represent sequenced ruminant animals and model species: *C.hircus, O.aries, B.taurus, S.scrofa, E.caballus, C.familiaris* and *H.sapiens*. Only the longest transcript isoform for each gene was kept, and only proteins longer than 30 amino acids were considered. The Treefam

methodology[27] was used to define a gene family as a group of genes descending from a single gene in the common ancestor of the species. Firstly, we used blastp (e-value < 1e-7) to find all the homologous relationship among protein sequences of all species and solar (in-house software, version 0.9.6) was used to conjoin high-scoring segment paris (HSPs) between each pair of protein homologs; then, protein sequences similarity were assessed with bit-score, and protein genes are clustered into gene families by program hcluster_sg (an implementation include in the Treefam pipeline, version 0.5.0) with an algorithm analogous to average-linkage clustering with the parameters set to be “-w 10 -s 0.34 -m 500 -b 0.1”.

3.2.2 Construction of phylogenetic tree

Single copy genes defined as orthologous genes according to Treefam were chosen in this analysis. The analysis included multiple alignments of protein sequences for each gene family. A Bayesian estimation of phylogeny, implemented in the MrBayes software (http://mrbayes.sourceforge.net, version 3.1.2)[28] was used for reconstruction of the evolutionary relationship between speices. The BRMC approach was used to estimate the species divergence time using the program MCMCTREE (version 4), which was part of the PAML package[29]. We also used PhyML[30] to reconstruct the phylogenetic tree and NCBI taxonomy common tree database, which align the result of MrBayes and confirm our result.

3.2.3 Expansion and contraction of gene families

Based on the phylogenetic tree topology, CAFÉ (Computational Analysis of gene Family Evolution, version 2.1)[28], a tool for the statistical analysis of the evolution of the size of gene families based on stochastic birth and death model, was used to detect gene families expansion and contraction in human, dog, horse, cow and goat with the parameters “P-value threshold 0.05, number of random 10000 and search for the λ value”. Gene families with P-values lower than 0.01 were analyzed manually. For expansions, goat genes in the family were manually checked for the same function assignment. For contracting gene families, the species with the largest number of proteins was checked for the functional consistency of its proteins. Expansion/contraction families whose family members had different functional assignment were filtered.

3.3 Rapidly evolving gene analysis

We used PAML package[29] to identify lineage-specific rapidly evolving genes in wild goat and domestic goat by estimating the omega ratio (ω) of non-synonymous to synonymous substitutions to examine the selective constraints on candidate genes. Using a simplified Treefam pipeline[27], 11,847 high confident 1:1:1:1 orthologous genes in wild goat, domestic goat, cattle and human were identified, whose coding sequence was then aligned using prank[31] with default parameters. Poorly aligned region was trimed by trimAl[32] with parameters"-automated1". We estimated the ω ratio for the 11,847 orthologous genes in four species by specifying either wild goat or domestic goat as foreground branch. 411 genes in the branch of wild goat and 820 ones in the branch of domestic goat have significant elevated ω ratio (P value < 0.05 for likelihood ratio test), which are defined as rapidly evolving genes. Considering that not all sites in nucleotide sequence have identical selection pressure, we then used branch-site model of PAML to detect positive selected sites in genes. Genes having both elevated ω and positive selected sites were then defined as positive selected genes (PSGs). To further filter out the false positive results, we then check non-synomous mutations in the alignment to exclude false positive selected sites calling induced by gaps and misalignment. After manual check, 18 PSGs in wild goat (Supplementary Table S12) and 52 PSGs in domestic goat (Supplementary Table S13) (P value < 0.05 for likelihood ratio test) were identified. Alignments of these PSGs are available now in Additional file2.

Configure file for branch model：
--runmode 0
--model 0 for null and 2 for alternative
--seqtype 1
--CodonFreq 2
--NSsites 0
--icode 0
--fix_kappa 0
--kappa 2
-fix_omega 0
--omega 1

Configure file for branch-site model
--runmode 0
--model 2
--seqtype 1

--CodonFreq 2
--NSsites 2
--icode 0
--fix_kappa 0
--kappa 2
--fix_omega 1 for mull and 0 for alternative
--omega 1

4 Supplementary Reference

1. Ye J, Fang L, Zheng H, Zhang Y, Chen J, Zhang Z, Wang J, Li S, Li R, Bolund L *et al*: **WEGO: a web tool for plotting GO annotations**. *Nucleic Acids Res* 2006, **34**(Web Server issue):W293-297.
2. Chang TC, Yang Y, Retzel EF, Liu WS: **Male-specific region of the bovine Y chromosome is gene rich with a high transcriptomic activity in testis development**. *Proceedings of the National Academy of Sciences of the United States of America* 2013, **110**(30):12373-12378.
3. Li R, Fan W, Tian G, Zhu H, He L, Cai J, Huang Q, Cai Q, Li B, Bai Y *et al*: **The sequence and de novo assembly of the giant panda genome**. *Nature* 2010, **463**(7279):311-317.
4. Marçais G, Kingsford C: **A fast, lock-free approach for efficient parallel counting of occurrences of k-mers**. *Bioinformatics* 2011, **27**(6):764-770.
5. Li R, Zhu H, Ruan J, Qian W, Fang X, Shi Z, Li Y, Li S, Shan G, Kristiansen K *et al*: **De novo assembly of human genomes with massively parallel short read sequencing**. *Genome Res* 2010, **20**(2):265-272.
6. Li R, Yu C, Li Y, Lam T-W, Yiu S-M, Kristiansen K, Wang J: **SOAP2: an improved ultrafast tool for short read alignment**. *Bioinformatics* 2009, **25**(15):1966-1967.
7. Parra G, Bradnam K, Korf I: **CEGMA: a pipeline to accurately annotate core genes in eukaryotic genomes**. *Bioinformatics* 2007, **23**(9):1061-1067.
8. Harris RS: **Improved pairwise alignment of genomic DNA**: ProQuest; 2007.
9. Kent WJ: **BLAT--the BLAST-like alignment tool**. *Genome Res* 2002, **12**(4):656-664.
10. Benson G: **Tandem repeats finder: a program to analyze DNA sequences**. *Nucleic Acids Res* 1999, **27**(2):573-580.
11. Xu Z, Wang H: **LTR_FINDER: an efficient tool for the prediction of full-length LTR retrotransposons**. *Nucleic Acids Res* 2007, **35**(Web Server issue):W265-268.
12. Stanke M, Keller O, Gunduz I, Hayes A, Waack S, Morgenstern B: **AUGUSTUS: ab initio prediction of alternative transcripts**. *Nucleic Acids Res* 2006, **34**(Web Server issue):W435-439.
13. Burge C, Karlin S: **Prediction of complete gene structures in human genomic DNA**. *J Mol Biol* 1997, **268**(1):78-94.
14. Allen JE, Majoros WH, Pertea M, Salzberg SL: **JIGSAW, GeneZilla, and GlimmerHMM: puzzling out the features of human genes in the ENCODE regions**. *Genome biology* 2006, **7 Suppl 1**:S9 1-13.
15. Majoros WH, Pertea M, Salzberg SL: **TigrScan and GlimmerHMM: two open source ab initio eukaryotic gene-finders**. *Bioinformatics* 2004, **20**(16):2878-2879.
16. Birney E, Clamp M, Durbin R: **GeneWise and Genomewise**. *Genome Res* 2004, **14**(5):988-995.
17. Haas BJ, Delcher AL, Mount SM, Wortman JR, Smith RK, Jr., Hannick LI, Maiti R, Ronning CM, Rusch DB, Town CD *et al*: **Improving the Arabidopsis genome annotation using maximal transcript alignment assemblies**. *Nucleic Acids Res* 2003, **31**(19):5654-5666.
18. Trapnell C, Williams BA, Pertea G, Mortazavi A, Kwan G, van Baren MJ, Salzberg SL, Wold BJ, Pachter L: **Transcript assembly and quantification by RNA-Seq reveals unannotated transcripts and isoform switching during cell differentiation**. *Nat Biotech* 2010, **28**(5):511-515.
19. Kim D, Pertea G, Trapnell C, Pimentel H, Kelley R, Salzberg SL: **TopHat2: accurate alignment of transcriptomes in the presence of insertions, deletions and gene fusions**. *Genome biology* 2013, **14**(4):R36.
20. Trapnell C, Williams BA, Pertea G, Mortazavi A, Kwan G, van Baren MJ, Salzberg SL, Wold BJ, Pachter L: **Transcript assembly and quantification by RNA-Seq reveals unannotated transcripts and isoform switching during cell differentiation**. *Nat Biotechnol* 2010, **28**(5):511-515.
21. Elsik CG, Mackey AJ, Reese JT, Milshina NV, Roos DS, Weinstock GM: **Creating a honey bee consensus gene set**. *Genome biology* 2007, **8**(1):R13.
22. Camacho C, Coulouris G, Avagyan V, Ma N, Papadopoulos J, Bealer K, Madden TL: **BLAST+: architecture and applications**. *BMC Bioinformatics* 2009, **10**:421.
23. Bairoch A, Apweiler R: **The SWISS-PROT protein sequence database and its supplement TrEMBL in 2000**. *Nucleic Acids Res* 2000, **28**(1):45-48.
24. Zdobnov EM, Apweiler R: **InterProScan – an integration platform for the signature-recognition methods in InterPro**. *Bioinformatics* 2001, **17**(9):847-848.
25. Ashburner M, Ball CA, Blake JA, Botstein D, Butler H, Cherry JM, Davis AP, Dolinski K, Dwight SS, Eppig JT

et al: **Gene Ontology: tool for the unification of biology**. *Nat Genet* 2000, **25**(1):25-29.
26. Kanehisa M, Goto S: **KEGG: kyoto encyclopedia of genes and genomes**. *Nucleic Acids Res* 2000, **28**(1):27-30.
27. Li H, Coghlan A, Ruan J, Coin LJ, Heriche JK, Osmotherly L, Li R, Liu T, Zhang Z, Bolund L *et al*: **TreeFam: a curated database of phylogenetic trees of animal gene families**. *Nucleic Acids Res* 2006, **34**(Database issue):D572-580.
28. Huelsenbeck JP, Ronquist F: **MRBAYES: Bayesian inference of phylogenetic trees**. *Bioinformatics* 2001, **17**(8):754-755.
29. Yang Z: **PAML 4: phylogenetic analysis by maximum likelihood**. *Mol Biol Evol* 2007, **24**(8):1586-1591.
30. Guindon S, Dufayard JF, Lefort V, Anisimova M, Hordijk W, Gascuel O: **New algorithms and methods to estimate maximum-likelihood phylogenies: assessing the performance of PhyML 3.0**. *Syst Biol* 2010, **59**(3):307-321.
31. Löytynoja A, Goldman N: **Phylogeny-Aware Gap Placement Prevents Errors in Sequence Alignment and Evolutionary Analysis**. *Science* 2008, **320**(5883):1632-1635.
32. Capella-Gutiérrez S, Silla-Martínez JM, Gabaldón T: **trimAl: a tool for automated alignment trimming in large-scale phylogenetic analyses**. *Bioinformatics* 2009, **25**(15):1972-1973.
